# Single-cell analysis of HIV expression and integration sites reveals robust viral expression across diverse chromatin environments

**DOI:** 10.1128/spectrum.00670-26

**Published:** 2026-06-30

**Authors:** Yun Ma, Jackson J. Peterson, David M. Margolis, Edward P. Browne

**Affiliations:** 1 Department of Microbiology and Immunology, UNC Chapel Hill 318275, Chapel Hill, North Carolina, USA; 2 UNC HIV Cure Center, UNC Chapel Hill 2331, Chapel Hill, North Carolina, USA; 3 Department of Medicine, UNC Chapel Hill 214908, Chapel Hill, North Carolina, USA; Karolinska Institutet, KarolStockholm, Sweden

**Keywords:** single cell, latency, HIV

## Abstract

Entry of HIV into latency is likely determined by a combination of factors, including stochastic fluctuations in the viral Tat protein during infection, as well as the transcriptomic phenotype of the host cell. Determining the impact of the proviral integration site on viral expression and latency has been challenging, in part due to difficulty in measuring the integration site and viral expression from the same cell. To investigate the influence of the HIV integration site on HIV expression, we analyzed a combined scRNA-seq/scATAC-seq data set from 117,610 HIV-infected primary CD4 T cells. We used the scATAC-seq data to recover HIV integration site information from 1,530 cells and correlated this information with viral RNA reads in the scRNA-seq data. We observed that, overall, HIV expression did not differ, depending on the genomic features of viral integration, such as genic vs non-genic, intron vs exon, and forward vs reverse orientation. Furthermore, we found that there was no significant difference in HIV expression across 15 distinct chromatin compartments. Additionally, single-cell expression analysis of HIV integration target genes revealed frequent (~5% of infections) insertional activation of host cell gene expression by HIV. This insertional activation occurred almost exclusively when HIV was integrated in the same orientation as the host cell gene and occurred as a result of integration within diverse positions across a gene body. These findings suggest that, overall, HIV expression is relatively robust to the genomic context of the HIV integration site and that HIV frequently upregulates expression of integration site genes during infection.

**IMPORTANCE:**

HIV integrates into the host genome during viral replication. In this study, we examine the impact of the integration site on HIV expression. We find that HIV expression is largely robust to variation in the genomic location of integration. We also find that HIV integration can often dramatically upregulate expression of host genes that HIV integrates into. Insertional activation of host cell genes could have important implications for HIV persistence during therapy and for pathogenesis. These findings provide new insight into how the virus HIV and the host cell interact and affect each other during viral replication.

## INTRODUCTION

Since its introduction in the 1990s, antiretroviral therapy (ART) has transformed human immunodeficiency virus (HIV) from a fatal illness into a manageable chronic condition (1). ART blocks multiple steps of the viral life cycle, suppressing viral replication and reducing both disease burden and transmissibility. However, viral rebound typically occurs when ART is interrupted, indicating the persistence of a long-lived, replication-competent viral reservoir (2–4). As a result, people with HIV require lifelong therapy. This persistence is due to HIV latency, where integrated proviruses enter a transcriptionally silent state, evading immune detection and drugs targeting HIV (5). Like other retroviruses, HIV reverse-transcribes its single-stranded RNA genome into double-stranded DNA and integrates into the host genome (6). A fraction of these proviruses becomes transcriptionally silent within infected cells, forming a latent reservoir that can reactivate and fuel rebound upon ART discontinuation (7). With a half-life of approximately 44 months under ART, the latent reservoir poses a major barrier to viral eradication (8, 9). Thus, understanding the mechanisms that govern HIV latency is essential for developing HIV cure strategies.

HIV integrates into the host genome in a non-random fashion, favoring transcriptionally active regions (6, 10). Integration places the provirus under the regulatory control of host chromatin, including histone modifications that influence gene expression. For example, H3K36me3 is associated with actively transcribed genes and mediates binding of LEDGF, a host factor that tethers HIV integrase to active chromatin (11, 12). By contrast, marks like H3K9me3 are enriched in heterochromatin and associated with transcriptional silencing. Together, these epigenetic marks define local transcriptional environments that may influence HIV expression and latency.

On the other hand, HIV proviral integration can influence host gene regulation to support viral persistence. When the provirus inserts within or near regulatory elements, it can perturb local transcriptional programs and, in some cases, drive clonal expansion of infected cells (13). Previous studies have shown that HIV can act as an enhancer-like element for nearby host genes, increasing chromatin accessibility and altering transcriptional output (14). Notably, integrations within genes such as *STAT3*, *STAT5B*, and *BACH2* have been associated with elevated host gene expression and subsequent T-cell outgrowth (15, 16). In the case of *STAT3*, activation depends on both the orientation of the provirus and its insertion within the first intron, underscoring the importance of genomic context (17). These examples demonstrate that proviral integration can dysregulate host genes in ways that contribute to reservoir persistence and pathogenesis.

To better understand how integration site (IS) features shape both viral and host gene expression, we analyzed single-cell ATAC and RNA sequencing data from HIV-infected CD4^+^ T cells. Specifically, we examined HIV IS location (intron, exon, or intergenic), local chromatin states, and insertion orientation relative to host gene transcription. Our results confirm that HIV preferentially integrates into transcriptionally active genes and that orientation is largely random. Surprisingly, we found that HIV expression is relatively robust across a range of integration site environments, suggesting limited sensitivity to local chromatin context. Moreover, we also observed that HIV frequently activates host gene expression when integrated in the same transcriptional direction, a phenomenon that appears to depend on both the location and orientation of the insertion site and may be generalizable across many genes. Together, our findings shed new light on how HIV proviral integration interacts with the host genome, contributing to the understanding of latency and reactivation.

## RESULTS

### Identification of HIV integration sites from single-cell ATAC-seq data

**Fig 1:**
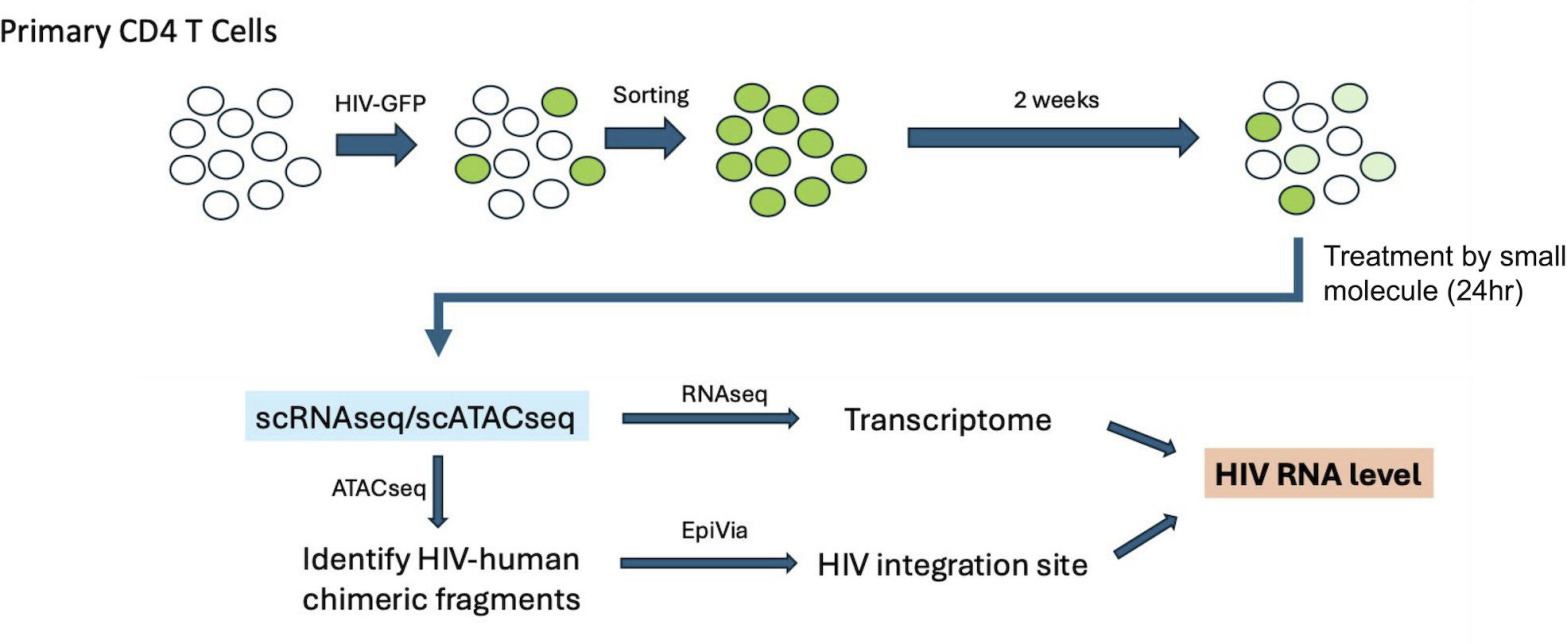
Schematic representation showing HIV infection, selection, and sequencing of single cells. Briefly, primary CD4 T cells from five different donors were activated and then infected with a GFP-expressing reporter strain of HIV. After 2 weeks of culture, a subset of the samples was treated with small molecules for 24 h, then the cells were profiled with a combined scRNA-seq/scATAC-seq protocol. Chimeric HIV–human fragments were then identified from the ATAC-seq data using epiVIA. Cellular transcriptomes were also derived and used to determine viral RNA expression for each cell.

The influence of the genomic environment near HIV integration sites (HIV ISs) on HIV viral expression, including chromatin features and proviral transcriptional orientation, has not been fully examined. To investigate this, we leveraged two previously published single-cell RNA-seq/ATAC-seq data sets which we generated from primary CD4^+^ T cells (from five independent donors) that were infected with a GFP-expressing reporter strain of HIV (18, 19). Following infection, the cells were maintained in culture for an additional 2–3 weeks, during which they returned to a resting state and downregulated HIV expression. For donors 1 and 2, the cell population underwent additional flow sorting to enrich for latently infected (GFP−) cells. Some cultures were then treated with small-molecule compounds for 24 h to assess their ability to reverse latency (Fig. 1). In the untreated cells and cells treated with latency-reversing agent (LRA) iBET151, approximately 6% of cells remained GFP+ (Fig. S1). In contrast, treatment with the LRA prostratin or suberoylanilide hydroxamic acid (SAHA) increased the proportion of fluorescent cells to 15% (18). Some samples were stimulated with 500 nM Δ-9-tetrahydrocannabinol (THC) or control vehicle (0.01% methanol) for 6 h, which had no effect on viral gene expression (19).

Given that the ATAC-seq data contain reads that map to integrated proviruses, we speculated that these data would also include reads that contained HIV–human junction points, thereby revealing the HIV integration sites in infected cells. To identify ISs from these data, we aggregated scRNA-seq/scATAC-seq data from a total of 16 HIV-infected CD4^+^ T-cell samples we have previously reported (18, 19), including five different donors (Table S1). We then used the epiVIA pipeline established by Wang et al. (20) to analyze paired-end ATAC sequencing data for HIV IS. In cases where the host–virus junction appeared in one sequencing read, the integration site could be directly identified. Otherwise, it was inferred when the two ends of a paired segment mapped to different organisms (termed “pair chimeric,” Fig. 1).

To validate that the identified HIV ISs were not PCR recombination artifacts, we examined the position and orientation of the HIV-aligned ends in pair-chimeric reads relative to the viral genome. Notably, in ~95% of chimeric fragments, the HIV-aligned reads localized within the viral long terminal repeats (LTRs) and were oriented toward the provirus termini (either 5′ or 3′). This non-random directional bias supports the conclusion that the captured HIV IS are unlikely to be PCR artifacts (Fig. S2A). Additionally, the frequency of HIV–host chimeric fragments was markedly higher than that of baseline host–host chimeric fragments (Fig. S2B). At first, chromosome 2 appeared to have an unusually high recombination rate, which was driven by reads mapping to the long non-coding RNA LINC000486. This same locus was previously identified as an artifact-prone region by Wang et al. (20) and automatically masked in their epiVIA pipeline. After removing reads aligned to LINC000486, the recombination rate for chromosome 2 became comparable to that of other host chromosomes. Finally, because donor sex was not specified, reads mapping to chromosome Y were excluded from analysis. In total, we analyzed 117,610 single cells, of which approximately 40% contained ATAC-seq fragments that map to HIV, and among these, we identified HIV IS in 1,530 cells (1.3%). We hereafter refer to the subset of cells with identified HIV integration sites as the IS+ population.

### HIV expression distribution is influenced by treatment and batch effects

Some of the infected samples used in this study were exposed to small molecules before profiling (SAHA, prostratin, iBET151, or THC), making treatment differences a potential confounding variable that could impact our results. For instance, SAHA and prostratin are known latency reversal agents (LRAs) that upregulate HIV expression (18). Consistent with previous reports, we observed that HIV expression distributions in SAHA- and prostratin-treated samples differed from those in DMSO controls (Fig. S3B). In addition to treatment effects, batch effects are another important consideration, as single-cell expression matrices are highly sensitive to technical variation (21). This is evident in Fig. S3A and B, where unstimulated samples 1 and 2 exhibit a distinct expression distribution compared to unstimulated samples 3–5, in both total RNA and HIV expression, likely reflecting a combination of batch effects and biological noise (Fig. S3D). To address these sources of variation, we applied the Seurat integration pipeline to harmonize mRNA expression values across samples (22). After integration, expression distributions appeared more consistent and approximately normal across all samples (Fig. S3E and G). To preserve the biological integrity of the data, we use the direct output of Seurat integration without additional transformations, such as *z*-scoring or fold change. Because Seurat already corrects for sequencing depth and technical variation, further processing may introduce artificial scaling and distort true biological signals (22). We therefore present the integrated expression distributions directly (Fig. S3H). Additional approaches such as DecontX (23) for ambient RNA correction were also assessed, which do not have a significant impact on the data distribution. To maintain the native data structure, further correction was not performed.

Neither donor variation (Fig. S3F) nor small-molecule treatment (Fig. S3C) appeared to strongly affect HIV integration frequency, as the proportion of HIV IS cells in most conditions was comparable to that of the overall combined data set. An exception was observed for unstimulated donor 1, where the fraction of HIV IS+ cells was approximately twofold lower than that of all cells in the same condition, potentially reflecting random variation in assay performance. In summary, the overall similarity in donor-specific proportions between all cells and HIV IS+ cells supports the interpretation that donor variation does not strongly influence integration site recovery.

Overall, the Seurat integrated HIV gene expression follows an approximately normal distribution with a right skew and a high density at zero, consistent with the sparse nature of single-cell data. For clarity, we will refer to the Seurat-integrated gene expression values as “normalized gene expression” to distinguish between Seurat integration (which corrects for technical variation) and HIV integration (the biological process of HIV provirus incorporation into the host genome). These normalized values are unitless, as described above. For additional interpretability, we also include comparisons using unprocessed raw counts.

### IS+ cells exhibit a similar transcriptomic profile to IS− cells

**Fig 2:**
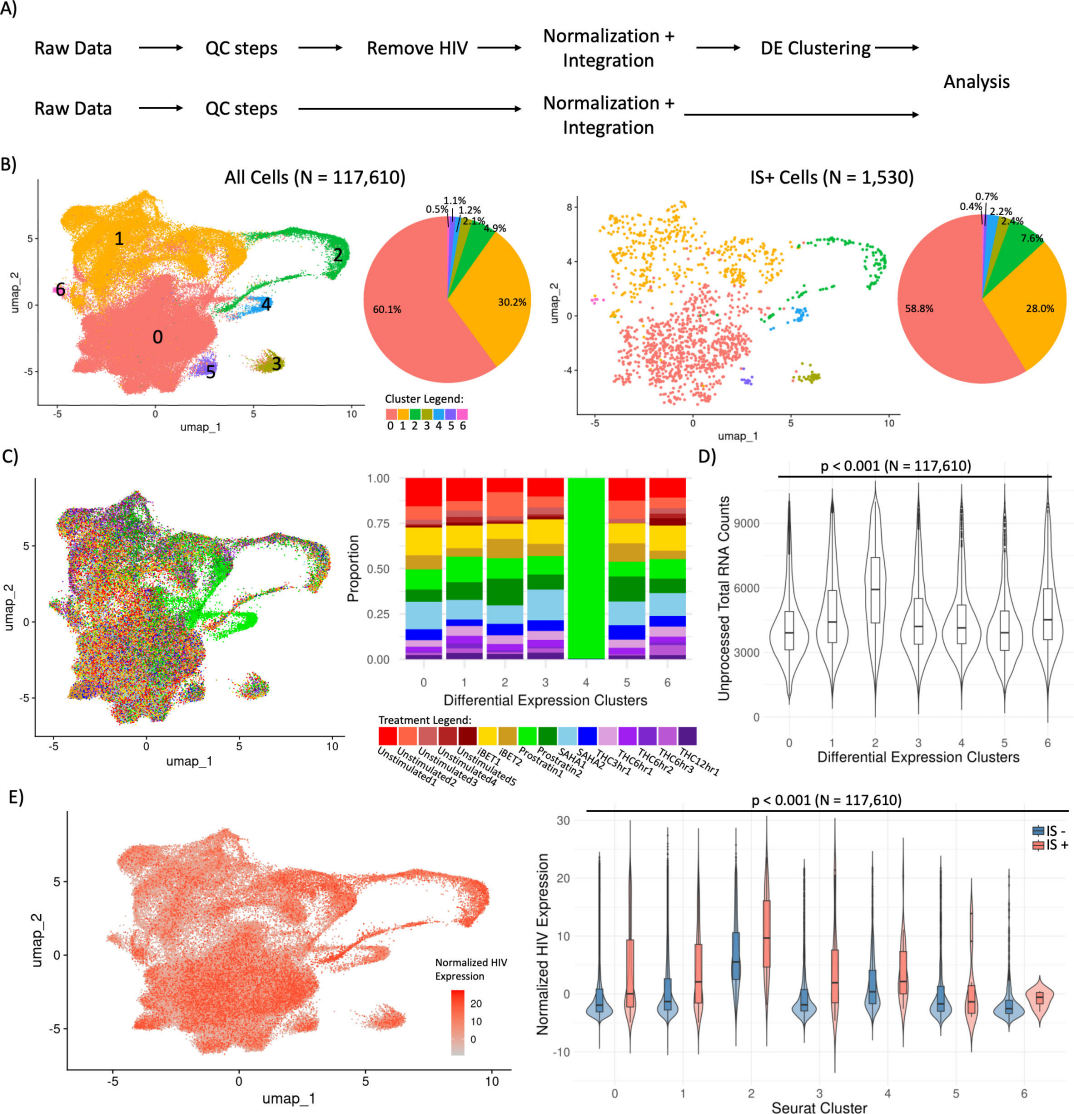
IS+ cells have an expression profile similar to IS− cells. (**A**) Schematic of the data processing pipeline. Normalized HIV expression and clustering results were generated from independent copies of the raw single-cell RNA-seq data to prevent HIV transcripts from driving clustering. (**B**) UMAP visualization of all cells (*N* = 117,610) and the IS+ subset (*N* = 1,530), colored by differential expression clusters. The accompanying pie chart shows the proportion of cells from each cluster within the population. (**C**) UMAP plot of all cells colored by treatment condition. The bar plot shows the distribution of treatment conditions across clusters. (**D**) Combined box plot and violin plot showing total mRNA content per cell across clusters. The Kruskal–Wallis test indicates significant differences across groups (*P* < 0.001, *N* = 117,610). (**E**) UMAP visualization of normalized HIV expression in all cells. The accompanying box plot and violin plot display the distribution of normalized HIV expression across clusters, stratified by IS status. Kruskal–Wallis tests show significant differences across clusters for both IS− and IS+ populations (*P* < 0.001 for each). Blue represents IS− cells; orange represents IS+ cells.

To investigate whether the host transcriptome is associated with the recovery of an IS, we performed differential expression (DE)-based clustering on Seurat-integrated scRNA-seq data. To ensure that variability in HIV expression did not drive clustering, we performed clustering on a version of the transcriptome with HIV expression excluded (Fig. 2A). We found that the overall distribution of IS+ cells (*N* = 1,530) across transcriptional clusters closely mirrored that of the total cell population (*N* = 117,610), indicating that the IS+ cells represent a largely random sampling of the host cells at the transcriptomic level (Fig. 2B). To assess whether the host cell transcriptional environment influences HIV expression, we first examined the distribution of treatment conditions across clusters to ensure that clustering was not confounded by treatments. The only exception to this was cluster 4, which was enriched for prostratin-treated cells, though this group comprised only 1.2% of the total population and had no significant difference in HIV expression pattern (Fig. 2C). Notably, cluster 2 showed elevated total mRNA content (Fig. 2D; Fig. S4A). We confirmed the biological significance of this difference via ontology analysis of genes upregulated in this cluster, revealing enrichment for cell cycle-related pathways, particularly those active during DNA synthesis and mitosis onset (Fig. S4C, right panel). We also observed that HIV expression was elevated in this cluster regardless of whether cells were IS+ or IS− (Fig. 2E; Fig. S4D). In summary, DE-based clustering revealed that recovery of HIV integration sites from the data is not broadly associated with cellular gene expression patterns and that HIV expression is influenced by the host cell transcriptional environment, consistent with previous reports (24).

### IS+ cells are enriched for transcriptionally active proviruses

**Fig 3:**
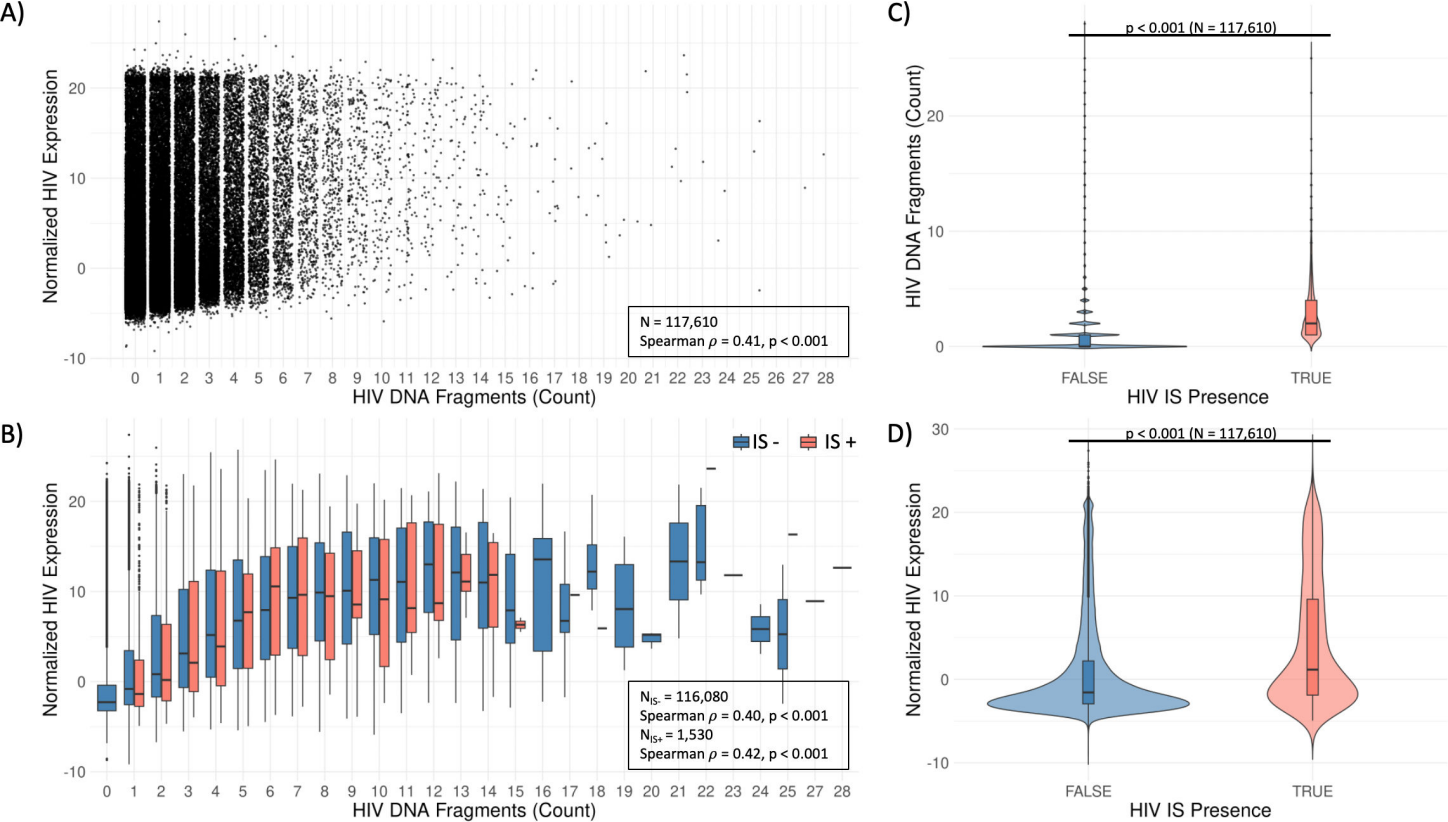
IS recovery is enriched for cells with ATAC-accessible and transcriptionally active proviruses. (**A**) Scatter plot showing the correlation between HIV RNA expression and the number of HIV DNA fragments (from ATAC-seq) per cell. Each point represents a single cell. The Spearman correlation coefficient is *ρ* = 0.41 (*P* < 0.001, *N* = 117,610). (**B**) Box plot illustrating the same correlation as in panel A, stratified by HIV IS status. Blue represents IS− cells (*ρ* = 0.41, *N* = 116,080), and orange represents IS+ cells (*ρ* = 0.43, *N* = 1,530). (**C**) Combined box plot and violin plot showing the distribution of HIV DNA fragments per cell in IS+ (orange) and IS− (blue) populations. IS+ cells have significantly more HIV DNA fragments (Wilcoxon test, *P* < 0.001). (**D**) Combined box plot and violin plot showing the distribution of HIV RNA expression per cell in IS+ (orange) and IS− (blue) populations. IS+ cells have significantly higher HIV expression (Wilcoxon test, *P* < 0.001).

We next examined HIV expression and accessibility in the IS+ population compared to the overall infected cell population. Because HIV ISs were identified using ATAC-seq, they were located within ATAC-accessible proviruses, which were open to host transcription factors and associated with elevated transcriptional activity. Based on this, we hypothesized that IS+ cells would exhibit higher levels of both HIV chromatin accessibility and RNA expression. Similar to what we have previously observed (18), we observed a positive correlation between the number of HIV DNA fragments (estimates of chromatin accessibility) and HIV RNA expression per cell (Spearman *ρ* = 0.41, Fig. 3A). This trend was observed across both treated and untreated samples, indicating it was not driven by small-molecule treatment (*ρ*_untreated_ = 0.38, *N*_untreated_ = 31,082; *ρ*_treated_ = 0.41, *N*_treated_ = 86,528). When stratifying cells by HIV IS status, both IS+ and IS− cells followed the same correlation pattern between HIV DNA and RNA levels, despite a large difference in sample sizes (Fig. 3B). As expected, no IS+ cells were found among those lacking HIV DNA fragments, since IS identification relies on the presence of HIV mapping DNA reads. Consistent with our hypothesis, IS+ cells contained significantly more HIV DNA fragments than IS− cells (Fig. 3C). Additionally, IS+ cells exhibited higher HIV RNA expression compared to IS− cells (Fig. 3D). These results confirm that the IS+ population is enriched for cells with transcriptionally active proviruses. Nevertheless, IS+ cells still displayed a wide range of expression levels from high to undetectable.

### HIV integration is enriched in transcriptionally active chromatin

**Fig 4:**
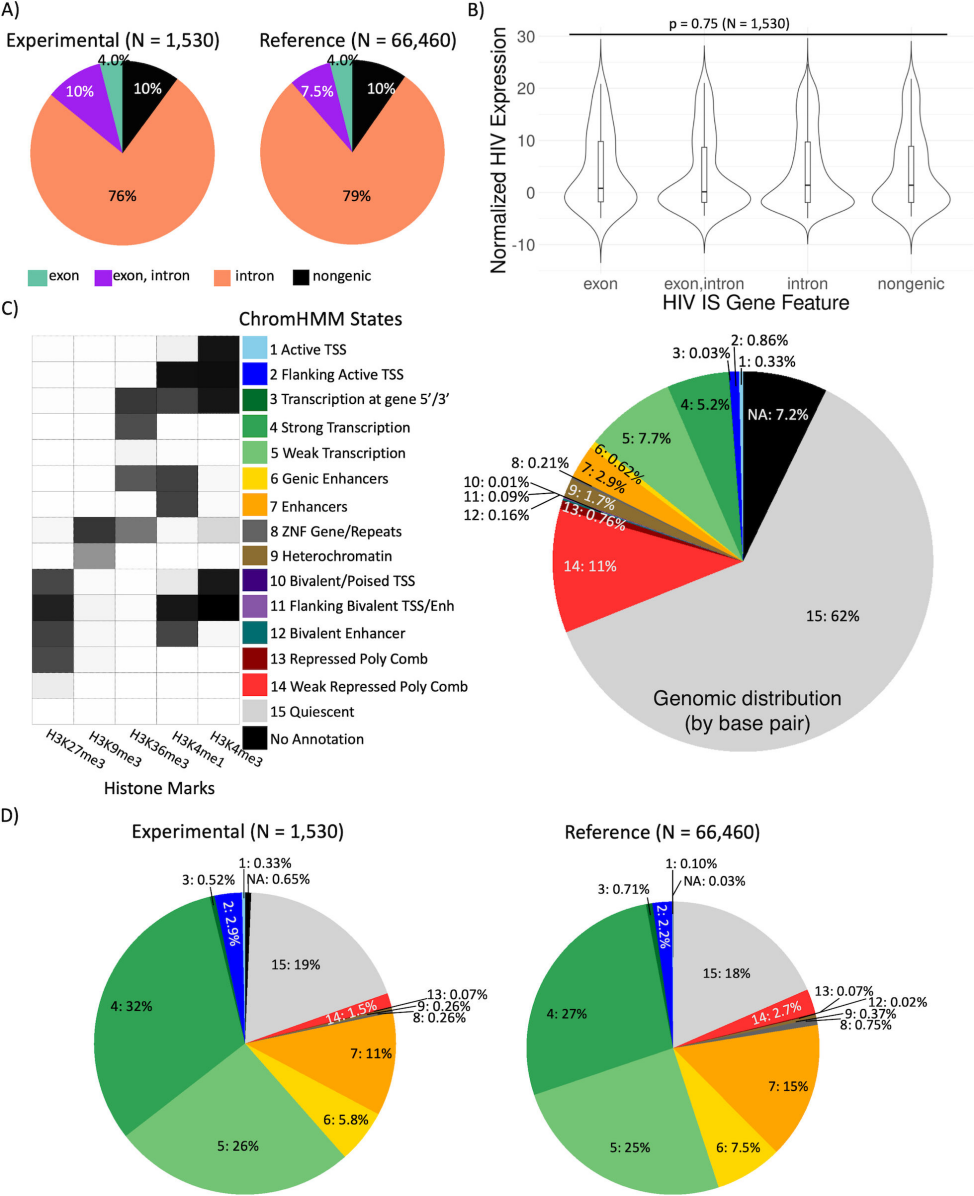
HIV integration is enriched in transcriptionally active chromatin. (**A**) Pie chart showing the distribution of HIV integration sites in our data set (“experimental”) located within introns, exons, or intergenic regions, compared to reference data from Coffin et al. (25). (**B**) Combined box plot and violin plot showing the distribution of HIV RNA expression in cells categorized by the genic feature surrounding the integration site. No significant difference was observed (Kruskal–Wallis test, *P* = 0.75; *N* = 1,530). (**C**) Characterization of chromHMM chromatin states, replotted from the emission parameters reported in the Roadmap Epigenomics Consortium (26). The heatmap displays the published emission probabilities for five histone marks across the 15 chromatin states, accompanied by the state abbreviations (bold) and short descriptions. The pie chart shows the proportion of the human genome (by base pairs) assigned to each state. (**D**) Pie charts showing the distribution of HIV integration sites across the 15 chromHMM-defined chromatin states, compared to the reference data set from Coffin et al. (25).

Having identified integration sites in a population of HIV-infected cells, we next investigated the genomic and epigenomic features surrounding HIV IS. Consistent with prior reports (25), HIV IS were enriched in genic regions (Fig. 4A). However, we found no significant difference in HIV expression between proviruses that were located within a gene (either in an exon or an intron) and those that were located in an intergenic region (*P* = 0.75, Fig. 4B). This observation held true across treatment conditions and when examining raw counts (Fig. S5A and B).

To examine the chromatin configuration surrounding HIV IS at a finer resolution, we utilized chromHMM, a hidden Markov model-based annotation system developed by the Roadmap Epigenomics Consortium (26, 26). This model was trained on five histone marks (H3K4me3, H3K4me1, H3K36me3, H3K27me3, and H3K9me3) at a 200 bp resolution across multiple cell types, including primary CD4^+^ T cells from peripheral blood (26). The chromHMM model assigns each 200 bp genomic region to 1 of 15 chromatin states based on histone mark emission densities, providing a reference for transcriptional and regulatory activities of the region (Fig. 4C). To ensure that this reference model was appropriate for our system, we evaluated the stability of the 15 chromHMM states across 14 cell types categorized as “blood and T cells” within the chromHMM project (Fig. S6A) (26). We found that state assignments were largely consistent across cell types both genome-wide (Fig. S6B) and within genic regions (Fig. S6C). Although some variability in chromatin state across CD4 T-cell subtypes was observed, it occurred primarily among adjacent or functionally similar chromatin states. A representative example involves states 4–7, which together account for more than 75% of the HIV integration sites in our data set. Within this group, only state 6 (genic enhancer) exhibits a self-transition probability below 50%; nevertheless, transitions among states 4–7 are almost entirely confined to this local neighborhood, with only a small fraction shifting to the quiescent state. This restricted switching pattern further supports the robustness of the 15-state model at this resolution (Fig. S6D).

To compare our IS population to a previously published data set of integration sites, we also examined 66,460 integration sites from HIV-infected peripheral blood CD4 T cells (25). We found that approximately 75% of HIV ISs were located within transcriptionally active chromatin states, specifically states 4, 5, 6, and 7 (Fig. 4D). This enrichment is significant, as those four states account for only 16.3% of the whole genome in base pairs (Fig. 4C). Furthermore, the chromatin state distribution of integration sites for our data strongly resembled the distribution from the reference set of integration sites (25), indicating that the integration sites recovered from our IS+ cell population are likely representative of the overall population with respect to the integration site pattern. In both our experimental data set and the reference, approximately 20% of HIV ISs were classified as “quiescent.” These sites lacked the five core histone marks, making their chromatin context inconclusive.

### HIV IS chromatin states do not influence HIV expression or accessibility

**Fig 5:**
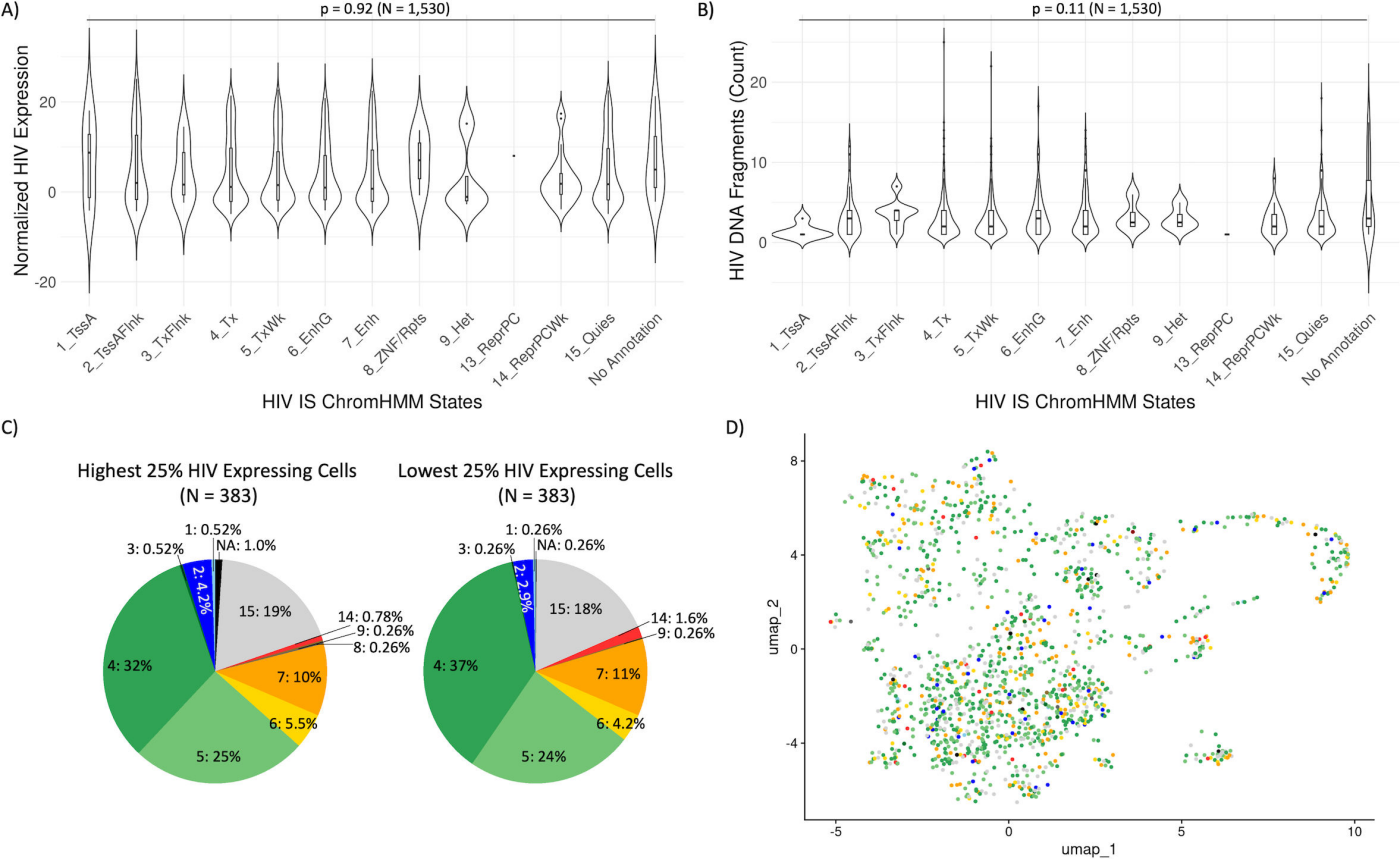
HIV IS chromatin states do not strongly influence HIV expression or accessibility. (**A**) Combined box plot and violin plot showing the distribution of HIV RNA expression across chromatin states of HIV integration sites. No significant differences were observed (Kruskal–Wallis test, *P* = 0.92; *N* = 1,530). (**B**) Combined box plot and violin plot showing the distribution of HIV DNA fragments (from ATAC-seq) per cell, categorized by HIV IS chromatin state. No significant differences were observed (Kruskal–Wallis test, *P* = 0.11; *N* = 1,530). (**C**) Pie charts showing the proportion of HIV integration sites assigned to each chromatin state, stratified by the highest and lowest quartiles of HIV-expressing cells (*N*_total_ = 1,530; *N*_quartile_ = 383 per group). (**D**) UMAP visualization of HIV IS+ cells colored by chromatin state, showing no strong spatial clustering of chromatin features across the transcriptomic landscape (*N* = 1,530). Enh, enhancer; EnhG, genic enhancer; Het, heterochromatin; Quies, quiescent; ReprPC, polycomb repressed; ReprPCWk, weakly polycomb repressed; TssA, active transcription start site; TssAFlnk, flanking active transcription start site; Tx, strongly transcribed gene; TxFlnk, flanking 5′ or 3′ region of an actively transcribed gene; TxWk, weakly transcribed gene; ZNF/Rpts, zinc finger repeats.

We next examined whether the chromatin state at the site of HIV integration influenced HIV expression. Overall, we found no significant correlation between HIV IS chromatin state and HIV RNA expression level (Kruskal–Wallis test, *P* = 0.92; Fig. 5A). This observation held true when expression levels were analyzed using unprocessed counts and stratified by treatment condition (Fig. S7A and B). However, a visual trend suggests that HIV integrated into state 9 (heterochromatin) tended to exhibit lower expression levels; this difference was not statistically significant in the Kruskal–Wallis test, likely due to the small number of observations (*N* = 4). Additional data would be needed to determine whether this trend is biologically meaningful. We also assessed whether the chromatin state influences the accessibility of HIV proviral DNA after integration, using the number of HIV-derived ATAC DNA fragments per cell as an approximation. Although the majority of HIV IS were in transcriptionally active regions, there was no significant difference in HIV DNA fragment counts across chromatin states (Fig. 5B). These data suggest that, once integrated, HIV provirus accessibility is not strongly influenced by the chromatin state of the integration site.

To further investigate whether chromatin context impacts viral activity, we compared the chromatin state distribution of HIV IS from the highest and lowest quartiles of HIV-expressing cells. We observed no significant differences in integration patterns between these groups (Fig. 5C). Additionally, mapping chromatin states onto a UMAP projection of host transcriptomes revealed no strong clustering, indicating that IS chromatin context is not tightly linked to overall host gene expression profiles (Fig. 5D).

Previous studies have suggested that HIV proviruses integrated into ZNF family genes, centromeric, or telomeric regions exhibit downregulated expression that promotes viral latency (27–30). However, exploratory analysis of this data set did not reveal significant differences in HIV expression between proviruses integrated into ZNF genes and those integrated elsewhere, likely due to the limited sample size of ZNF-integrated cells (*n* = 15 of 1,530 total IS+ cells). Notably, all ZNF-integrated proviruses were found exclusively in LRA-treated samples (iBET, prostratin, and SAHA), suggesting potential treatment-induced detection bias for ZNF integrated provirus. Similarly, only one cell with centromeric integration (on chromosome 11) was identified, containing a single HIV RNA copy with normalized HIV expression value of 0.27. No telomeric integrations were detected. The low discovery rate for integration sites in these regions may be attributed to ATAC sequencing bias toward accessible chromatin or mapping issues with repetitive centromeric and telomeric regions.

**Fig 6:**
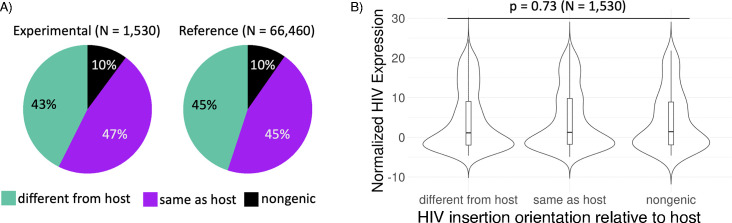
HIV insertion orientation does not influence HIV expression. (**A**) Pie charts showing the proportion of HIV integration sites oriented in the same or opposite transcriptional direction as the host gene, compared to Coffin et al. (25). (**B**) Combined box plot and violin plot showing HIV RNA expression stratified by HIV insertion orientation. No significant difference was observed (Kruskal–Wallis test, *P* = 0.73; *N* = 1,530).

We also sought to examine how the orientation of HIV integration relative to the host gene’s transcriptional direction affects viral expression. We observed that, for proviruses within host cell genes, HIV integration orientation appeared random with respect to the host gene (Fig. 6A), and orientation had no apparent impact on HIV RNA expression (Fig. 6B). This finding remained consistent when analyzed using unprocessed counts and stratified by treatment condition (Fig. S8).

Overall, these data suggest that HIV expression is largely independent of the genic context or orientation of its integration site and that the host chromatin state at the site of integration does not appear to strongly influence HIV accessibility and expression once integrated.

### HIV integration results in frequent insertional activation of host cell genes

We next examined whether HIV affects expression of the host cell gene into which it integrates. In our data set, we identified individual cases in which HIV integration appeared to potently upregulate expression of the host gene in a manner that was specific to both the orientation and location of the integration (Fig. S9), where upregulation is defined by comparing the expression of the HIV-integrated gene in each IS+ cell to the same gene’s expression in all other cells in the data set.

**Fig 7:**
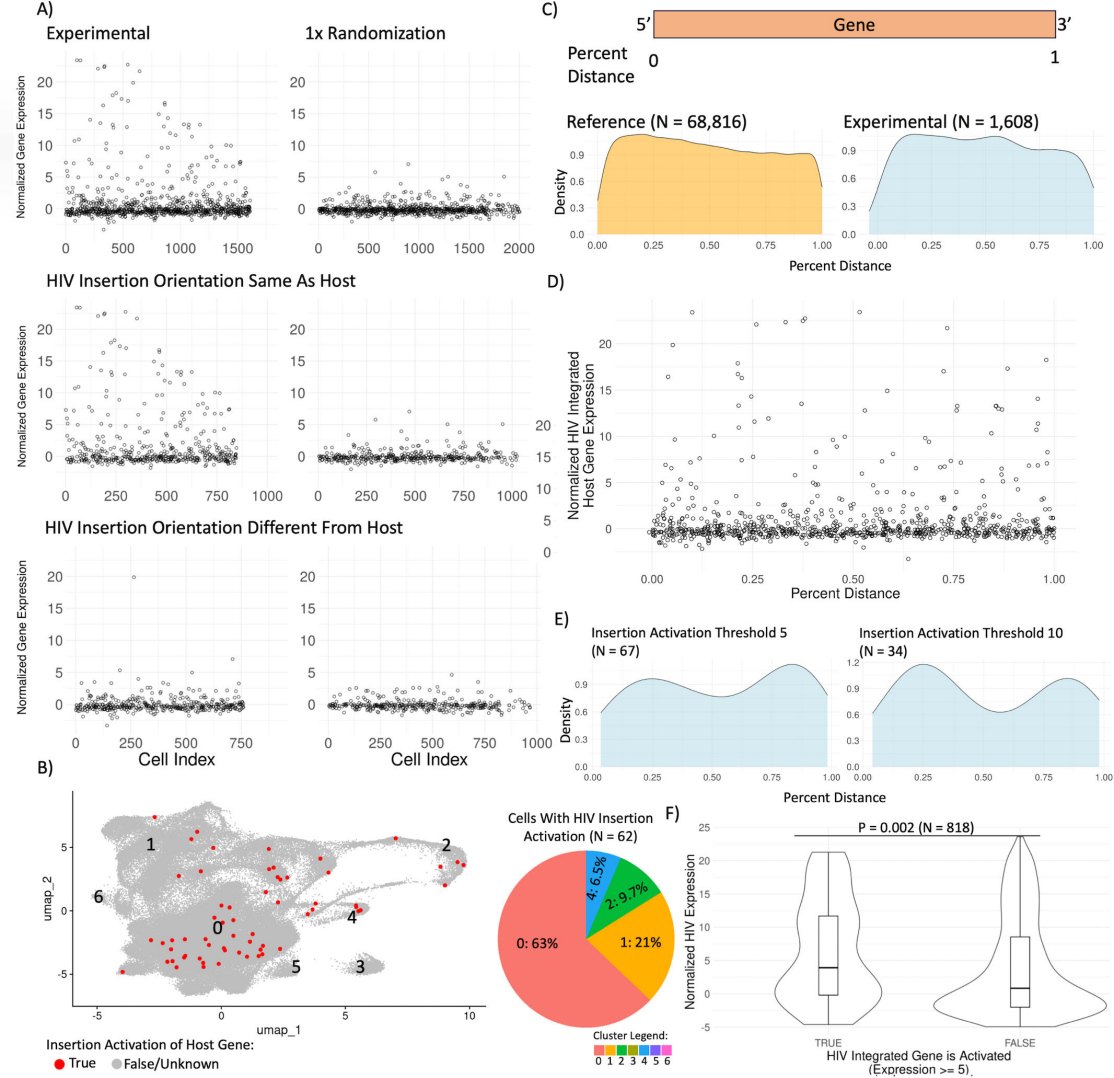
HIV integration promotes frequent insertional activation of host cell genes. (**A**) Scatter plots showing the expression of HIV-integrated host genes, compared to randomly generated integration sites. (Top row) All samples; (middle and bottom rows) stratified by HIV insertion orientation relative to the host gene. (**B**) UMAP projection of all cells, with HIV insertion-activated cells highlighted in red and the rest of the population in gray. The pie chart indicates the proportions of transcriptional clusters within the insertion-activated subset. (**C**) Schematic defining percent distance of HIV IS along the gene body, with accompanying density plot comparing IS distributions in the experimental data set and Coffin et al. (25). (**D**) Scatter plot showing the relationship between insertion site location and host gene activation. No significant monotonic trend was observed. (**E**) Density plots showing HIV IS distribution along genes at different thresholds of host gene upregulation (e.g., approximately ≥5 or ≥10 standard deviations above the mean). (**F**) Combined box plot and violin plot showing HIV RNA expression in cells with and without host gene insertional activation. HIV expression is significantly higher in the insertion-activated group (Kruskal–Wallis test, *P* = 0.002; *N* = 818).

To test whether this phenomenon is generalizable, we aggregated gene expression values across HIV-inserted genes in all IS+ cells. Because Seurat normalization and integration produce expression values that are approximately normally distributed with a mean near 0 and a standard deviation (SD) near 1 for each gene, direct comparisons across genes are valid. As a negative control, we also generated a simulated data set of randomly placed *in silico* HIV integration sites and performed the same analysis. We found that, in more than 5% of IS+ cells, expression of the integrated gene exceeded the mean expression of that gene across the population by a large margin (approximately more than 5 standard deviations from the mean, Table S2). This phenomenon was absent in the randomized data set, indicating a biological effect rather than a sampling artifact. Moreover, this insertional activation occurred almost exclusively when HIV was integrated in the same orientation as the host gene (Fig. 7A; Fig. S10). To control for potential confounding effects from globally high gene expression cells (e.g., DE cluster 2, identified earlier in Fig. 2D), we assessed the proportion of cluster 2 cells among insertion-activated cells. While insertion-activated cells were somewhat enriched for cluster 2 (9.7%) compared to all cells (4.9%) and IS+ cells (7.6%), the overall low proportion suggests that neither cluster 2 nor other clusters are a major driver of the observed insertional activation (Fig. 7B). We performed pathway analysis of the insertion-activated gene set using g:Profiler and Enrichr (31–34), but the two tools yielded inconsistent results, and the enriched categories showed weak adjusted *P* values (Tables S3 and S4). This likely reflects the limited number of insertion-activated genes identified in our data set (67 genes), which may be insufficient for robust enrichment analysis.

Next, we examined whether the position of the HIV integration site within the host gene affects the likelihood of insertional activation. HIV integrations were distributed broadly across gene bodies, with a modest enrichment near gene start sites (Fig. 7C). While no monotonic trend emerged between percent distance along the gene and activation likelihood, density plots using increasing thresholds for defining high expression revealed bimodal enrichment: integration near the 5′ and 3′ ends of genes was more likely to be associated with extreme host gene activation (approximately 5 SDs above the mean, Fig. 7D and E). This enrichment diminished at higher thresholds, potentially due to reduced sample size.

In addition, we observed a weak correlation between HIV expression in IS+ cells and the expression of the host gene into which it was integrated. Notably, HIV RNA levels were significantly higher in cells where insertional activation of the host gene occurred (*P* = 0.002, *N* = 818; Fig. 7F). This suggests that insertional activation of host genes is more frequent for highly expressed proviruses.

Together, these findings suggest that HIV integration can lead to frequent insertional activation of host genes, particularly when the provirus is in the same orientation as the gene. However, the locational specificity and precise details of these events remain unclear and will require further investigation to elucidate underlying mechanisms.

## DISCUSSION

The influence of local genomic features surrounding HIV ISs on both viral and host gene expression remains largely uncharacterized. In this study, we used single-cell ATAC-seq to identify HIV integration sites and correlated them with host and viral transcriptional profiles obtained from matched single-cell RNA-seq data. Our findings confirm that HIV preferentially integrates into transcriptionally active regions of the genome, consistent with previous bulk analyses (6, 10). Notably, HIV expression appeared relatively robust across diverse genomic contexts. In addition, we observed that HIV integration can potently upregulate the expression of host genes at the integration site, particularly when the provirus is inserted in the same transcriptional orientation as the host gene.

We identified HIV integration sites using the epiVIA pipeline (20) and validated them by confirming a higher frequency of host–HIV chimeric read pairs compared to host–host inter-chromosomal chimeras, which likely reflect PCR recombination artifacts. Additionally, HIV reads were consistently aligned to LTRs and oriented outward from the viral genome, a pattern suggesting genuine host–viral junctions in unsequenced parts of the DNA segment. Together, these features support the biological authenticity of the identified integration sites.

A major limitation of this approach, however, is its sensitivity. Despite successful infection being confirmed in all cells by fluorescence reporter and ~40% of cells containing detectable HIV DNA by ATAC-seq, we were able to confidently assign integration sites in only ~1% of cells. This discrepancy likely reflects the low probability of capturing a host–virus junction by random paired-end ATAC fragments. Moreover, it introduces interpretive ambiguity: the IS− population likely includes both cells lacking accessible integration sites and cells for which the integration status is inconclusive.

To address technical biases inherent to ATAC-seq, particularly the reliance on proviral accessibility (“ATAC bias”), we compared our data set with a reference set of HIV integration sites identified via nested PCR (25), which is independent of chromatin accessibility. We also applied Seurat integration (22) to mitigate batch effects and harmonize HIV expression across treatment conditions. After correction, small-molecule treatments and individuals had minimal impact on expression clustering, with the exception of a prostratin-driven cluster (cluster 4, ~1.2% of cells), which did not affect the overall HIV or host gene expression. By contrast, cluster 2 showed elevated total RNA levels and was enriched for genes associated with cell cycle phases between DNA synthesis and mitosis (Fig. S3C). While HIV expression was elevated in this cluster regardless of integration status, cluster 2 did not contribute disproportionately to insertional activation events. These findings suggest that although cell cycle-associated transcriptional activity may enhance HIV expression, it is largely independent of HIV integration site features.

Our analysis confirmed that HIV preferentially integrates into intronic regions of the genome. However, whether this preference results from an intrinsic affinity for introns or simply reflects the larger proportion of intronic sequences within genic regions remains inconclusive. Preliminary attempts to clarify this by comparing intronic and exonic base pair compositions yielded inconclusive results due to complications arising from overlapping genic annotations.

To further investigate the host genomic environment around HIV IS at a higher resolution, we applied the chromHMM model, which classifies 200 bp genomic windows based on five histone modification marks (12, 26). Our findings revealed a preferential integration of HIV into transcriptionally active chromatin states, consistent with previous reports (6, 11). We ruled out the possibility that this integration preference results from ATAC bias by comparing our data set with a reference data set (25) obtained from people with HIV. Despite differences in cell populations and sequencing methods, the proportions of HIV IS across genomic categories remained consistent, suggesting that HIV integration preferences observed are fundamental to its mechanism. However, our single-cell IS and gene expression analysis did not reveal a significant correlation between genomic features and HIV viral expression. Several factors may explain this observation. First, as mentioned earlier, ATAC-seq bias may inherently select for transcriptionally accessible sites. Second, while visual differences in HIV expression were observed between some genomic categories, statistical significance was not observed, potentially due to small sample sizes in some chromatin states (e.g., heterochromatin, where only four cells were identified). A more efficient IS identification method could improve statistical power and validate these trends. Third, it is possible that the chromatin state of the surrounding genome does not strongly dictate the provirus’s transcriptional accessibility. Given that chromHMM assigns chromatin states based on 200 bp windows and HIV is approximately 10 kb long, integration could alter or override the local chromatin landscape. Thus, we speculate that HIV expression may be inherently robust to the local chromatin environment around the integration site, with previous reports of silencing in transcriptionally inactive regions representing outlying cases (30).

One limitation of our approach is the extent to which the chromHMM reference model can be generalized to our system. Prior work has shown that HIV infection and exposure to small molecules can alter the host transcriptome and, by extension, potentially the chromatin landscape, raising concerns about applying a reference chromHMM model directly to our data (18). Due to the historical nature of the data set being analyzed in this study, obtaining matched ChIP-seq data were not possible, and combining our approach with single-cell ChIP-seq methods would be very technically challenging. Nevertheless, to address this limitation, we assessed the consistency of chromHMM annotations across 14 T-cell-related blood cell types included in the chromHMM project (26). We found that 9 of the 14 states are assigned consistently more than 50% of the time across cell types, and when states do change, transitions predominantly occur among neighboring or functionally related states. These patterns suggest that although the chromHMM reference model may not be a perfect match for our system, it is sufficiently robust to broad differences in transcriptional programs to serve as a practical reference.

Recent reports have demonstrated that intact proviruses are preferentially preserved in high repetitive chromatin regions during long-term ART and in elite controllers (27, 29). While we observed a modest trend toward lower expression in H3K9me3-rich chromatin compartments, this effect was not significant, possibly due to the low numbers of integration sites within our data set. Overall, our data suggested that HIV expression can be robust regardless of being in a seemingly repressive chromatin environment. Nevertheless, studies of HIV integration sites in clonal T-cell lines indicate diverse and stable patterns of expression for identical proviruses with different sites and orientations (35–38), arguing that integration site details can have an important impact on HIV expression. Our approach also may not fully account for integration events into highly repetitive regions of the genome, such as telomeres and centromeres, due to mapping issues during alignment to these regions, and an adapted experimental approach may be needed to address the behavior of these proviruses.

Finally, we also observed that HIV integration can frequently activate the expression of host genes. These insertional activation events appear to depend on the orientation of the provirus relative to the host gene and may also be influenced by the specific location of the integration site within the gene body. While orientation-dependent activation was clearly supported as it occurred almost exclusively when the provirus and host gene were aligned, we could not conclusively determine “within-gene” location specificity. This may reflect limitations in our current analytic approach, which uses percent distance along the gene as a positional indicator. A more precise definition involving regulatory elements could help resolve this question in future studies. Earlier work has shown that HIV can upregulate integration site genes (14–17). These reports focused largely on oncogenic targets, likely because they confer selective growth advantages to infected cells, while our results indicate that insertion-mediated activation is a broader and more frequent phenomenon than previously recognized.

To build on these findings, future work could focus on functionally characterizing the host genes activated by HIV and elucidating the downstream pathways they affect. This remains challenging because our approach is limited in ability to link functional outcomes for the cell to specific integration events. It is possible that insertional activation of some of these host genes could provide a growth or survival advantage to the cell, similar to what has been observed for STAT3 and STAT5B (15, 16). Another possibility is that insertional activation of host genes could contribute to immune dysfunction and pathogenesis by perturbing some aspect of immune homeostasis other than cell division. Another direction for future work will be to evaluate proviral intactness and examine its relationship to HIV expression and insertional activation. Prior studies suggest that approximately 60% of proviruses remain intact during single-round *in vitro* infection (39), raising the possibility that intactness may influence either the likelihood of proviral transcription or its ability to modulate nearby host gene expression.

In conclusion, our study provides key insights into the genomic landscape of HIV integration and its effects on host and viral transcription. We confirm that HIV preferentially integrates into transcriptionally active genic regions, a pattern that appears to be an intrinsic viral preference across diverse conditions. However, we did not observe a strong correlation between integration site features and viral expression, likely due to the intrinsic robustness of HIV transcription. Notably, our results suggest that HIV integration can induce frequent host gene upregulation in an orientation-dependent manner. Future work combining large data sets, refined regulatory mapping, and functional assays will be critical to fully elucidate how integration site characteristics influence HIV latency and reservoir dynamics.

## MATERIALS AND METHODS

### Data set acquisition and alignment of scATAC-seq and scRNA-seq data

The data sets analyzed in this study were obtained from the Gene Expression Omnibus under accession number GSE242997 (18), along with additional data previously reported in Ashokkumar et al. (19). Briefly, primary CD4^+^ T cells were isolated from the peripheral blood of HIV-seronegative donors, and cell purity was confirmed by flow cytometry (18). Cells were then activated with anti-CD3/CD28 beads for 3 days and infected with a GFP-encoding HIV strain (HIV-ΔGFP/Thy1.2) (18). Successfully infected (GFP+) cells were identified at 2 days post-infection and enriched by flow sorting. Following enrichment, the infected cells were cultured for a 2- to 3-week period to allow a subset of the cells to downregulate viral gene expression and become latently infected (GFP−). For donors 1 and 2, a latently infected (GFP−) cell population was then further enriched by a second round of flow sorting, then treated with the indicated compounds according to the protocol described in Ashokkumar et al. (18). For the Ashokkumar et al. data set (donors 3, 4, and 5), isolated and infected CD4^+^ T cells were co-incubated with matched peripheral blood mononuclear cells at a 1:3 ratio following their return to latency, and the mixed cell population was stimulated with 500 nM Δ9-THC or with the control vehicle (0.01% methanol) for various time periods and subsequently sequenced together (19). To delineate the CD4^+^ T-cell compartment within this mixed data set, an initial clustering analysis was conducted using the Seurat pipeline (22). Clusters exhibiting the highest levels of HIV transcripts were identified as HIV-infected CD4^+^ T cells and were selected for all downstream analyses.

Combined single-cell ATAC and RNA sequencing were performed using the 10× Genomics multiome platform, and libraries were sequenced with an Illumina NextSeq 2000 platform (18). Sequences were aligned using the 10× Genomics Cell Ranger ARC software. A custom human–HIV reference genome, incorporating the HIV-ΔGFP/Thy1.2 construct, was built with the mkref function (18). Alignment against this custom reference genome was performed using the cellranger-arc count function. For subsequent analyses, the resulting .bam files were used for chromatin accessibility information, while the filtered_feature_bc_matrix outputs were used for transcriptomic analyses.

### Identification of HIV integration site with epiVIA

Paired chimeric reads were identified in the .bam files from the alignment, defined as reads with two ends aligned to different chromosomes (in the alignment .bam file, these reads have a seventh column value that is not an “=” sign). The baseline paired chimeric rate was calculated by dividing the number of paired chimeric reads on a chromosome by the total number of reads aligned to that chromosome. HIV integration sites were identified following the epiVIA pipeline described by Wang et al. (20). The .bam files from scATAC-seq alignments served as inputs. Custom reference genome FASTA files, incorporating the human-HIV and HIV-dGFP/Thy1.2 sequences (18), were indexed using BWA (40) and used for integration site mapping. In cases where a cell had multiple HIV ISs, the whole cell was excluded from the study.

### Annotation of local chromatin structure by chromHMM

The chromHMM annotations, based on primary T-helper cells from peripheral blood (ID: E043) from the NIH Roadmap Epigenomics Consortium, were used to define chromatin states (12, 26). These annotations were generated using a 15-state hidden Markov model trained on five core histone marks (H3K4me3, H3K4me1, H3K36me3, H3K27me3, and H3K9me3) and provided as the E043_15_coreMarks_hg38lift_mnemonics_sorted.bed file (12, 26). HIV integration sites identified using epiVIA were processed and matched to these chromatin annotations. The GenomicRanges package in R was used to assign a chromatin state to each HIV integration site (41, 42). In cases where an HIV IS had two chromHMM states (because the HIV IS coordinates may be a range), a random one was retained.

### Annotation of the orientation of HIV provirus relative to host transcriptional direction

HIV-integrated genes were reannotated using the main 23 chromosomes from GENCODE v46 via the UCSC Table Browser (43–45), with gene symbols extracted from hg38.kgXref. The orientation of each HIV integration site, as determined by epiVIA, was compared to the orientation of the gene it integrated into and classified as either “same as host,” “different from host,” or NA when HIV was not integrated in a gene.

### Annotation of gene features around the HIV integration site

The GENCODE v46 reference described previously (44, 45) was used to define gene features. BED files for introns and exons were generated directly using the UCSC Table Browser (43–45). Non-genic regions were created by subtracting intron and exon intervals from the whole genome BED file using the bedtools subtract function (46). With the GenomicRanges package in R (41), each HIV integration site was assigned a feature type “exon,” “intron,” or “non-genic,” based on the interval containing it. If an HIV integration site was both intronic and exonic due to overlapping genes, it was categorized as “exon;intron.”

### Reference data set of HIV integration sites

The reference data set of HIV integration sites was obtained from the publicly accessible Retrovirus Integration Database v2.0 (25). It was lifted to the hg38 genome assembly using the hg19-to-hg38 chain file (44, 47) and annotated with the GENCODE v46 reference for compatibility with the experimental data (43). All integration features were annotated using the methods described above.

### Normalization and integration of single-cell gene expression data

The gene expression data were processed using the Seurat software suite (22). Doublets were identified and removed from each of the 16 samples using scDblFinder in R (48). The data were then normalized using Seurat’s SCTransform pipeline with default settings, following the “Using SCTransform in Seurat” tutorial (22). After normalization, the 16 samples were integrated following the “Introduction to scRNA-seq Integration” tutorial (22).

### Differential expression clustering of single cells based on gene expression data

A copy of the gene expression data, independent from the previous section, was made. After doublets were removed as described previously, HIV expression was removed from the expression matrix. Normalization and integration followed the same pipeline as before. The data were clustered using Seurat’s clustering pipeline, following the “Guided Clustering Tutorial” (22). The biological implications of clusters were assessed by browsing the list of significantly differentially expressed genes (log2 fold change ≥5) in Enrichr (32–34).

### Simulation of random HIV integration sites and integrated genes

To assess the influence of HIV integration events on host gene expression, randomly generated sites were compared with experimentally derived HIV integration sites. These random sites were distributed proportionally across intronic, exonic, and non-genic regions to match the observed distribution in the actual data set. Each random site was assigned a cell barcode and HIV orientation corresponding to the feature type (e.g., pseudo-exon sites were matched to the barcodes and orientations of real exon sites). No barcodes were reused in the 1:1 randomization process, ensuring an unbiased comparison. Randomization was performed using a fixed seed for reproducibility. The simulated sites were annotated with associated genes using the GENCODE v46 reference as described in the previous sections (43–45). Gene expression values for the corresponding cell barcodes were extracted from the actual Seurat object.

### Definition of HIV integration site percent distance in a gene

The relative position of an HIV integration site within a gene was measured using percent distance, where the start and end of the gene were defined as 0 and 1, respectively. The percent distance for each integration site was calculated using the following formula:


(1) $$\text{ Percent Distance }=\left\{ \begin{array}{ll} \frac{\text{ HIV IS - Gene Start }}{\text{ Gene End - Gene Start }} & \text{ if host gene orientation is " }+"\\ 1-\frac{\text{ HIV IS - Gene Start }}{\text{ Gene End - Gene Start }} & \text{ if host gene orientation is " }-" \end{array}\right.$$

### Statistical analysis

The statistical tests in this study were conducted in R (42). For comparisons among more than two groups, a Kruskal–Wallis test was used to identify differences (42), followed by a post hoc Dunn’s pairwise test when appropriate (49). For features with two groups, a Wilcoxon rank-sum test was performed (42). Correlations were calculated using the Spearman method (42). All graphics were generated using base R, ggplot2, LocusZoomR, or matplotlib in Python (42, 50–52).

## Data Availability

All code and data used in this analysis are available in the following Github directory: https://github.com/yunma1108/sc-HIV-Integration-Site. Data and code also available at the UNC Dataverse: https://doi.org/10.15139/S3/0XHJC5,

## SUPPLEMENTAL MATERIAL

Supplemental figures spectrum.00670-26-s0001.pdf Figures S1 to S10.

Supplemental file legends spectrum.00670-26-s0002.docx Legends for Figures S1 to S10 and Tables S1 to S4.

Table S1 spectrum.00670-26-s0003.xlsx Sample information, including the number of cells collected for each donor and treatment condition.

Table S2 spectrum.00670-26-s0004.xlsx List of all HIV-integrated genes. HIV insertion-activated genes are highlighted in green (threshold: Seurat integrated gene expression ≥ 5).

Table S3 spectrum.00670-26-s0005.xlsx Enrichment analysis for HIV insertion-activated genes performed using g:Profiler (31). The second tab includes the search parameters used.

Table S4 spectrum.00670-26-s0006.xlsx Enrichment analysis for HIV insertion-activated genes performed using Enrichr (32-34).

ASM does not own the copyrights to Supplemental Material that may be linked to, or accessed through, an article. The authors have granted ASM a non-exclusive, world-wide license to publish the Supplemental Material files. Please contact the corresponding author directly for reuse.
