# Supplementary material for: Single-cell analysis of HIV expression and integration sites reveals robust viral expression across diverse chromatin environments
Source: Microbiol Spectr. 2026 Jun 30;14(8):e00670-26. doi: 10.1128/spectrum.00670-26 (PMC13436327; doi:10.1128/spectrum.00670-26)
Supplement: Supplementary file 1 [file spectrum.00670-26-s0001.pdf]

Figure S1

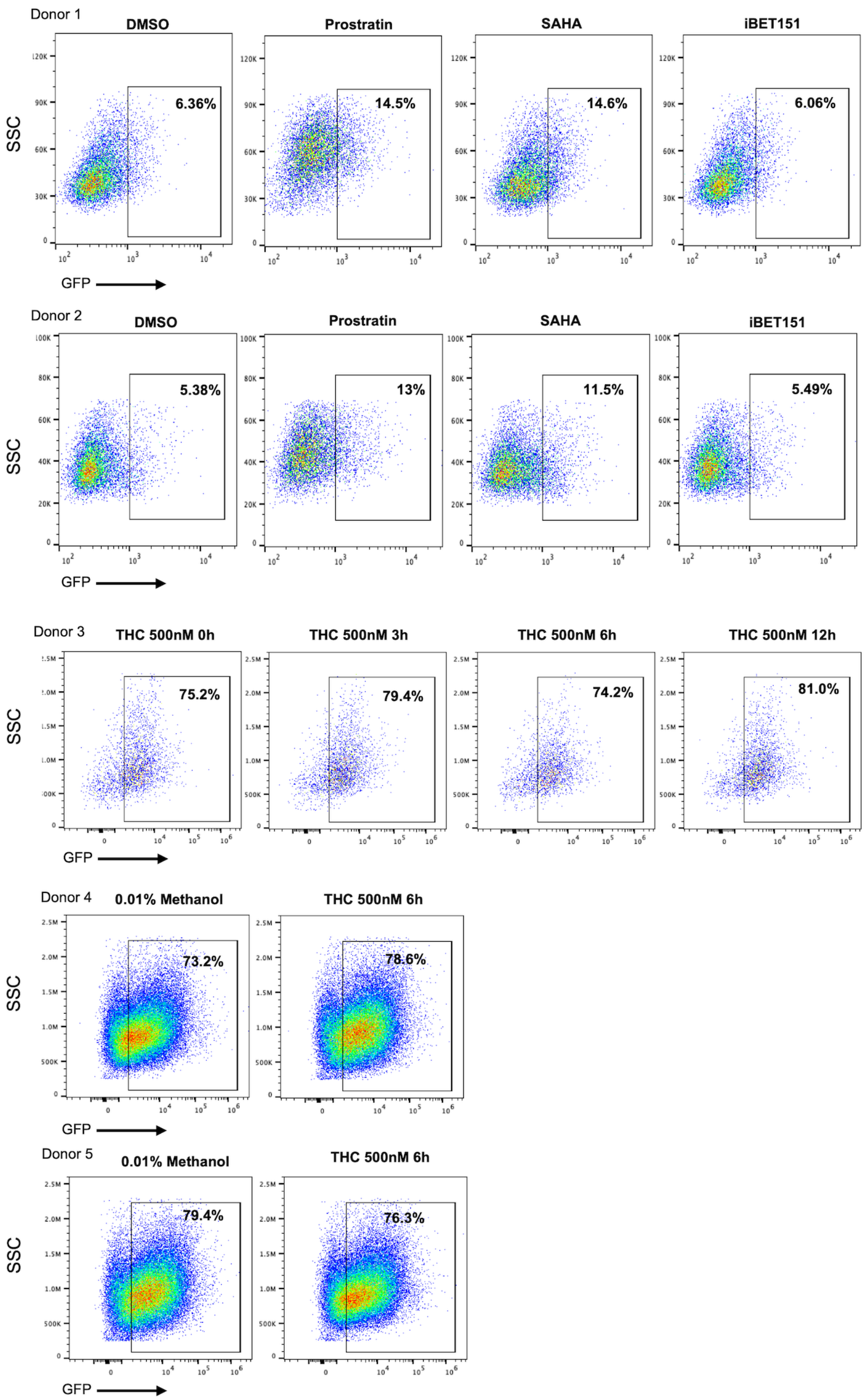

Donor 1
DMSO
Prostratin
SAHA
iBET151
6.36%
14.5%
14.6%
6.06%
SSC
GFP
Donor 2
DMSO
Prostratin
SAHA
iBET151
5.38%
13%
11.5%
5.49%
SSC
GFP
Donor 3
THC 500nM 0h
THC 500nM 3h
THC 500nM 6h
THC 500nM 12h
75.2%
79.4%
74.2%
81.0%
SSC
GFP
Donor 4
0.01% Methanol
THC 500nM 6h
73.2%
78.6%
SSC
GFP
Donor 5
0.01% Methanol
THC 500nM 6h
79.4%
76.3%
SSC
GFP

Figure S2

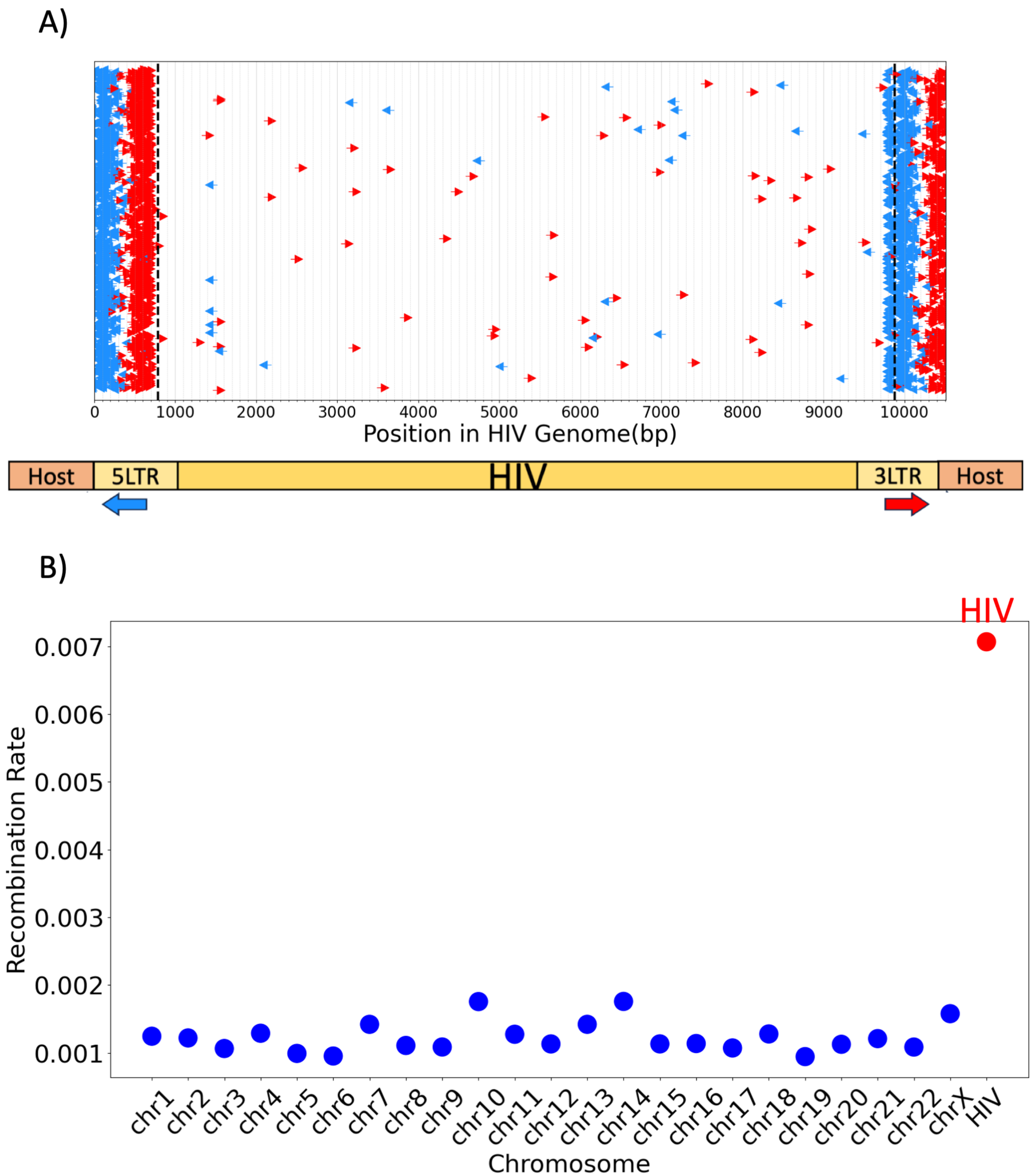
A)
0
1000
2000
3000
4000
5000
6000
7000
8000
9000
10000
Position in HIV Genome(bp)
Host
5LTR
HIV
3LTR
Host
B)
HIV
0.007
0.006
0.005
0.004
0.003
0.002
0.001
Recombination Rate
chr1
chr2
chr3
chr4
chr5
chr6
chr7
chr8
chr9
chr10
chr11
chr12
chr13
chr14
chr15
chr16
chr17
chr18
chr19
chr20
chr21
chr22
chrX
HIV
Chromosome

Figure S3

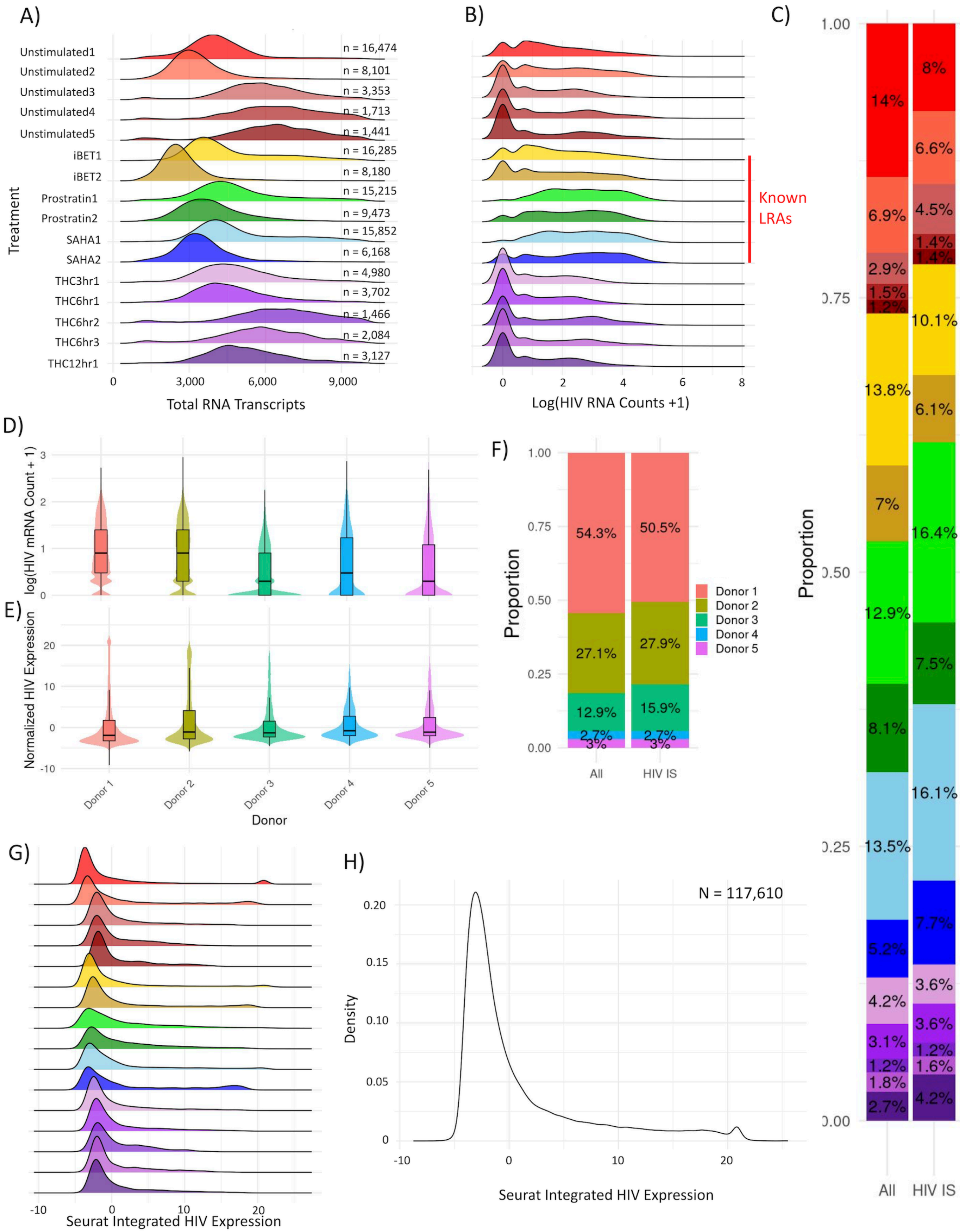

A)
Unstimulated1
Unstimulated2
Unstimulated3
Unstimulated4
Unstimulated5
iBET1
iBET2
Prostratin1
Prostratin2
SAHA1
SAHA2
THC3hr1
THC6hr1
THC6hr2
THC6hr3
THC12hr1
Treatment
n = 16,474
n = 8,101
n = 3,353
n = 1,713
n = 1,441
n = 16,285
n = 8,180
n = 15,215
n = 9,473
n = 15,852
n = 6,168
n = 4,980
n = 3,702
n = 1,466
n = 2,084
n = 3,127
0
3,000
6,000
9,000
Total RNA Transcripts
B)
Known LRAs
0
2
4
6
8
Log(HIV RNA Counts +1)
C)
1.00
0.75
0.50
0.25
0.00
Proportion
14%
6.9%
2.9%
1.5%
1.2%
13.8%
7%
12.9%
8.1%
13.5%
5.2%
4.2%
3.1%
1.2%
1.8%
2.7%
8%
6.6%
4.5%
1.4%
1.4%
10.1%
6.1%
16.4%
7.5%
16.1%
7.7%
3.6%
3.6%
1.2%
1.6%
4.2%
All
HIV IS
D)
log(HIV mRNA Count + 1)
3
2
1
0
E)
Normalized HIV Expression
20
10
0
-10
Donor 1
Donor 2
Donor 3
Donor 4
Donor 5
Donor
F)
1.00
0.75
0.50
0.25
0.00
Proportion
54.3%
27.1%
12.9%
2.7%
3%
50.5%
27.9%
15.9%
2.7%
3%
All
HIV IS
Donor 1
Donor 2
Donor 3
Donor 4
Donor 5
G)
-10
0
10
20
Seurat Integrated HIV Expression
H)
N = 117,610
0.20
0.15
0.10
0.05
0
Density
-10
0
10
20
Seurat Integrated HIV Expression

Figure S4

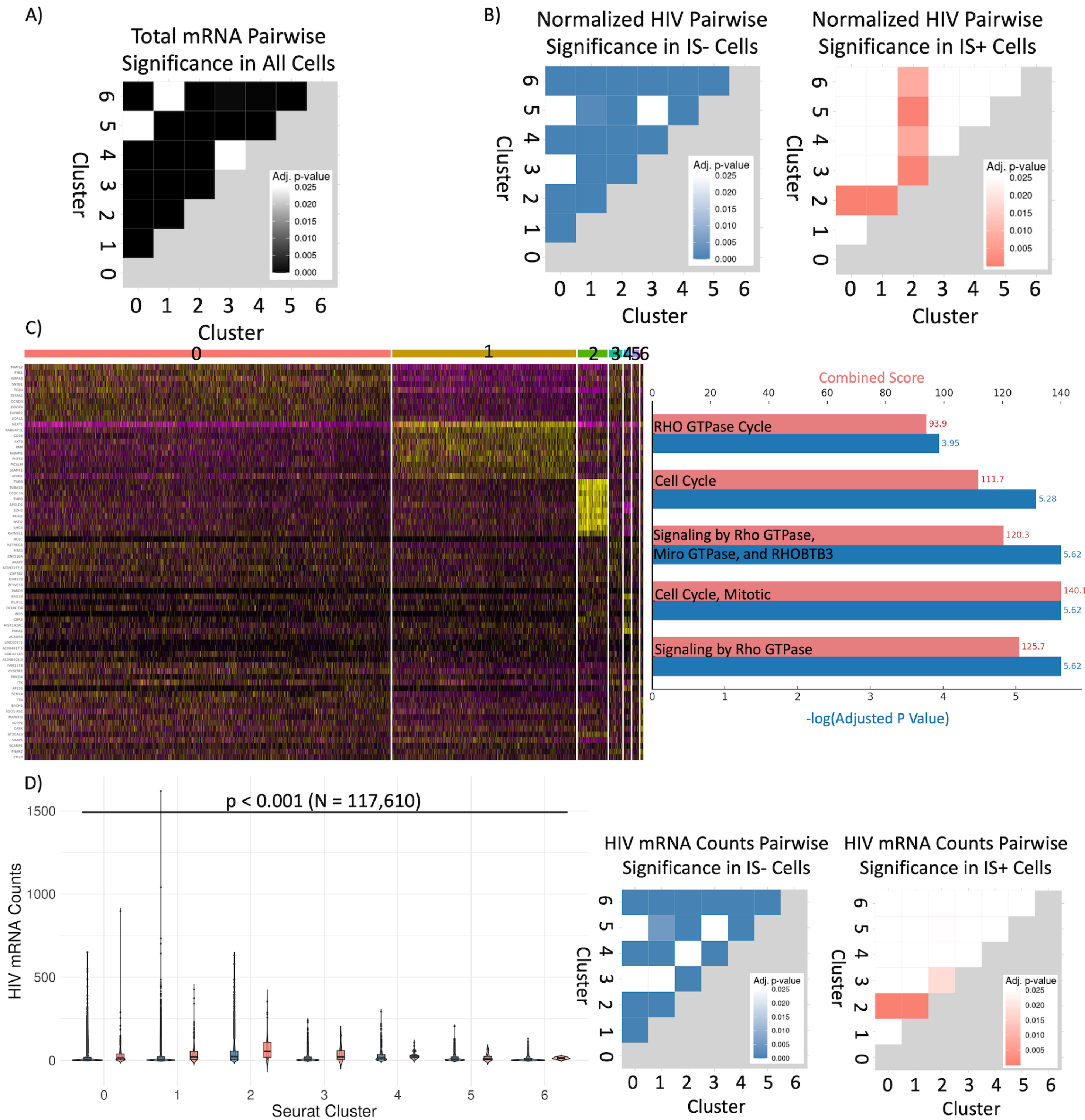

A)
Total mRNA Pairwise Significance in All Cells
Cluster
Adj. p-value
0.025
0.020
0.015
0.010
0.005
0.000
0 1 2 3 4 5 6
B)
Normalized HIV Pairwise Significance in IS- Cells
Normalized HIV Pairwise Significance in IS+ Cells
Cluster
C)
0 1 2 3 4 5 6
Combined Score
RHO GTPase Cycle 93.9 3.95
Cell Cycle 111.7 5.28
Signaling by Rho GTPase, Miro GTPase, and RHOBTB3 120.3 5.62
Cell Cycle, Mitotic 140.1 5.62
Signaling by Rho GTPase 125.7 5.62
-log(Adjusted P Value)
D)
p < 0.001 (N = 117,610)
HIV mRNA Counts
Seurat Cluster
HIV mRNA Counts Pairwise Significance in IS- Cells
HIV mRNA Counts Pairwise Significance in IS+ Cells

Figure S5

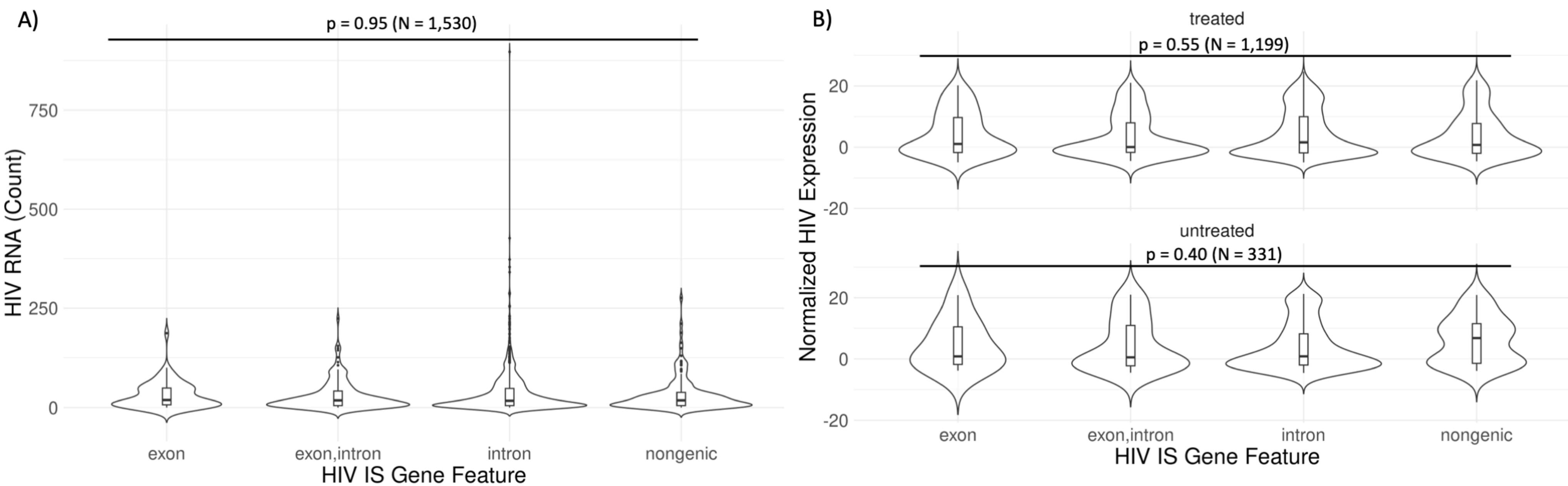
A)
p = 0.95 (N = 1,530)
HIV RNA (Count)
750
500
250
0
exon
exon,intron
intron
nongenic
HIV IS Gene Feature
B)
treated
p = 0.55 (N = 1,199)
untreated
p = 0.40 (N = 331)
Normalized HIV Expression
20
0
-20
20
0
-20
exon
exon,intron
intron
nongenic
HIV IS Gene Feature

Figure S6

A)

Cell Types
Primary mononuclear cells (PB)
Primary T cells from primary blood (PB)
Primary T cells effector/memory enriched (PB)
Primary T cells from cord blood
Primary T regulartory cells (PB)
Primary T helper cells (PB)
Primary T helper cells PMA-I stimulated
Primary T helper 17 cells PMA-I stimulated
Primary T helper memory cells (PB)
Primary T helper memory cells (PB, replicate)
Primary T CD8+ memory cells (PB)
Primary T helper naive cells (PB)
Primary T helper naive cells (PB, replicate)
Primary T CD8+ naive cells (PB)

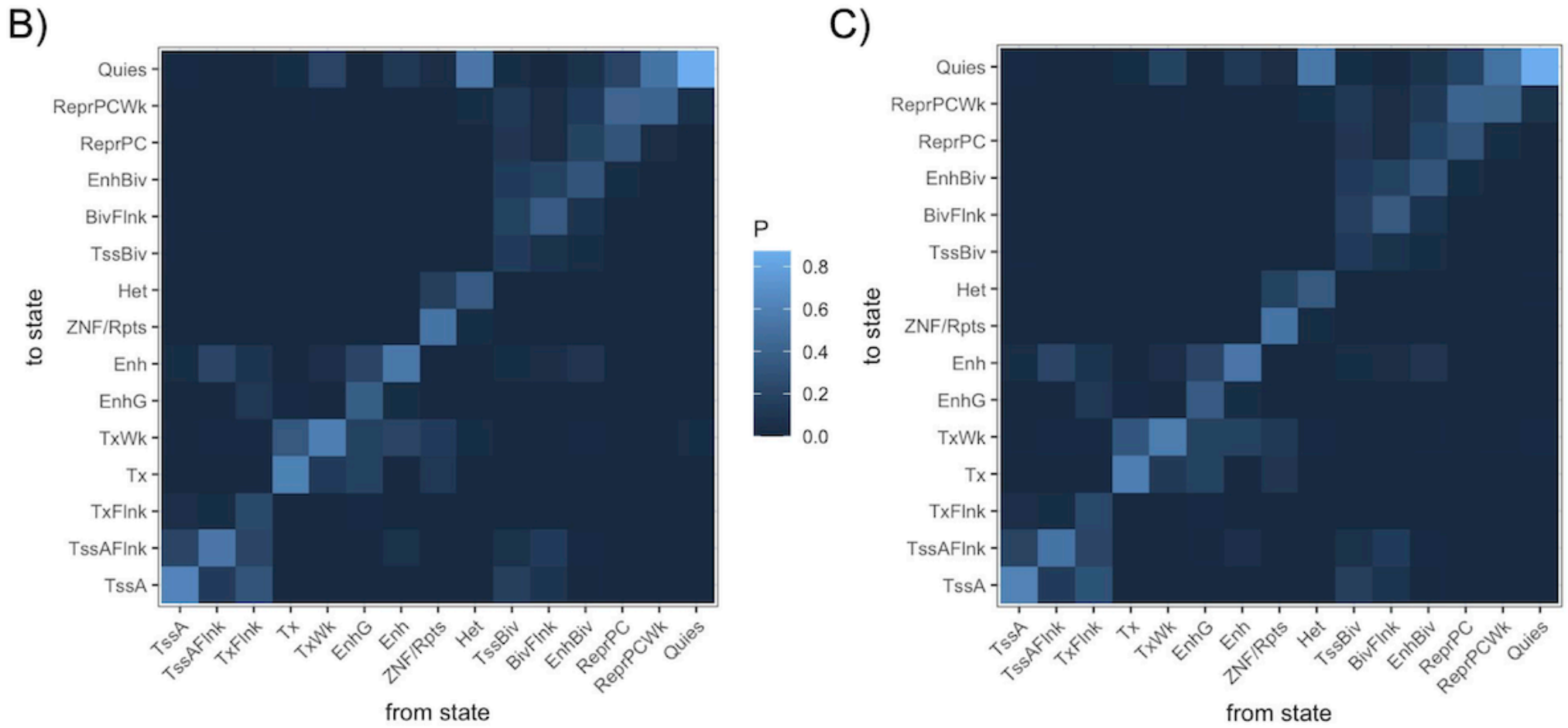

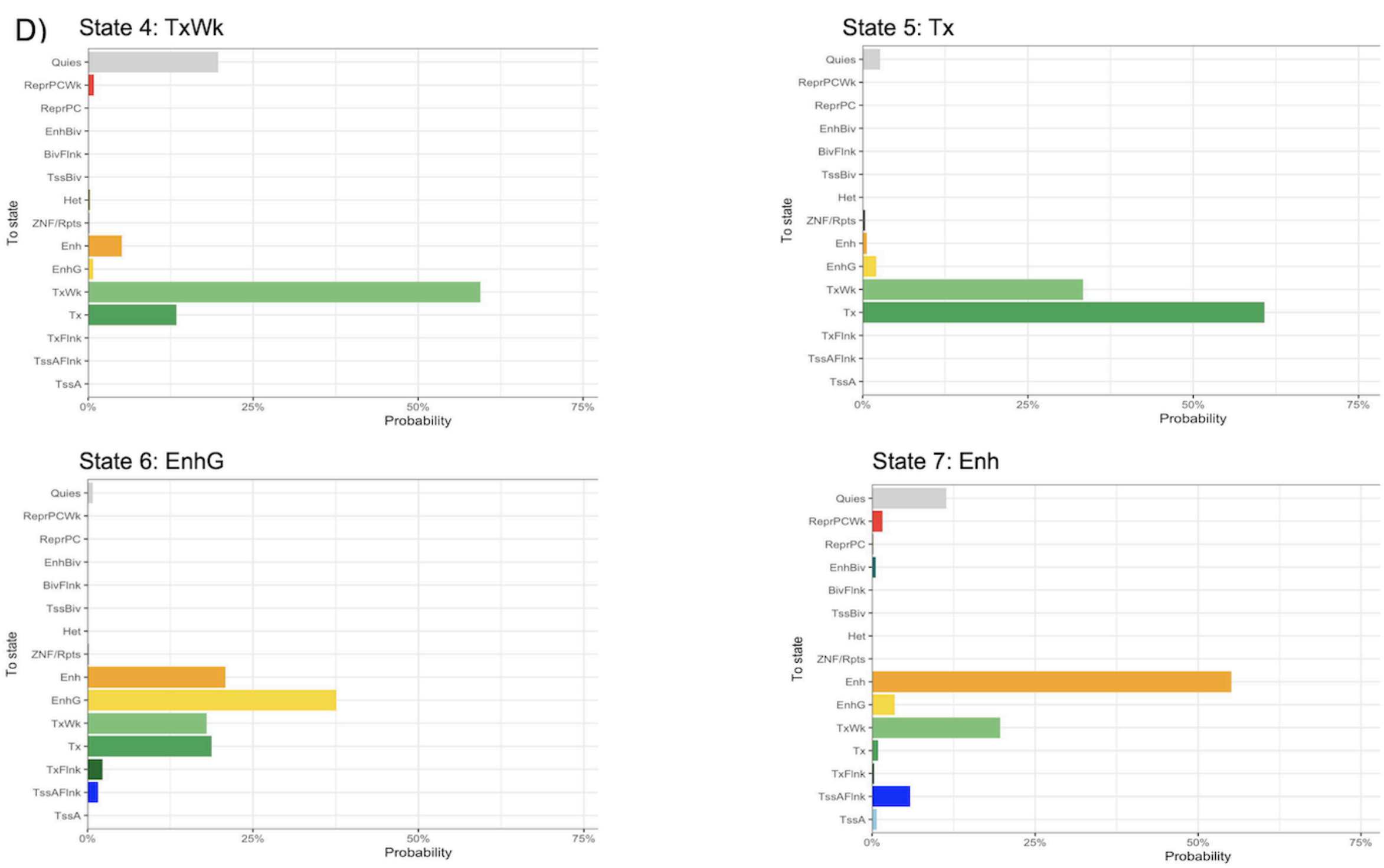

Figure S7

A)

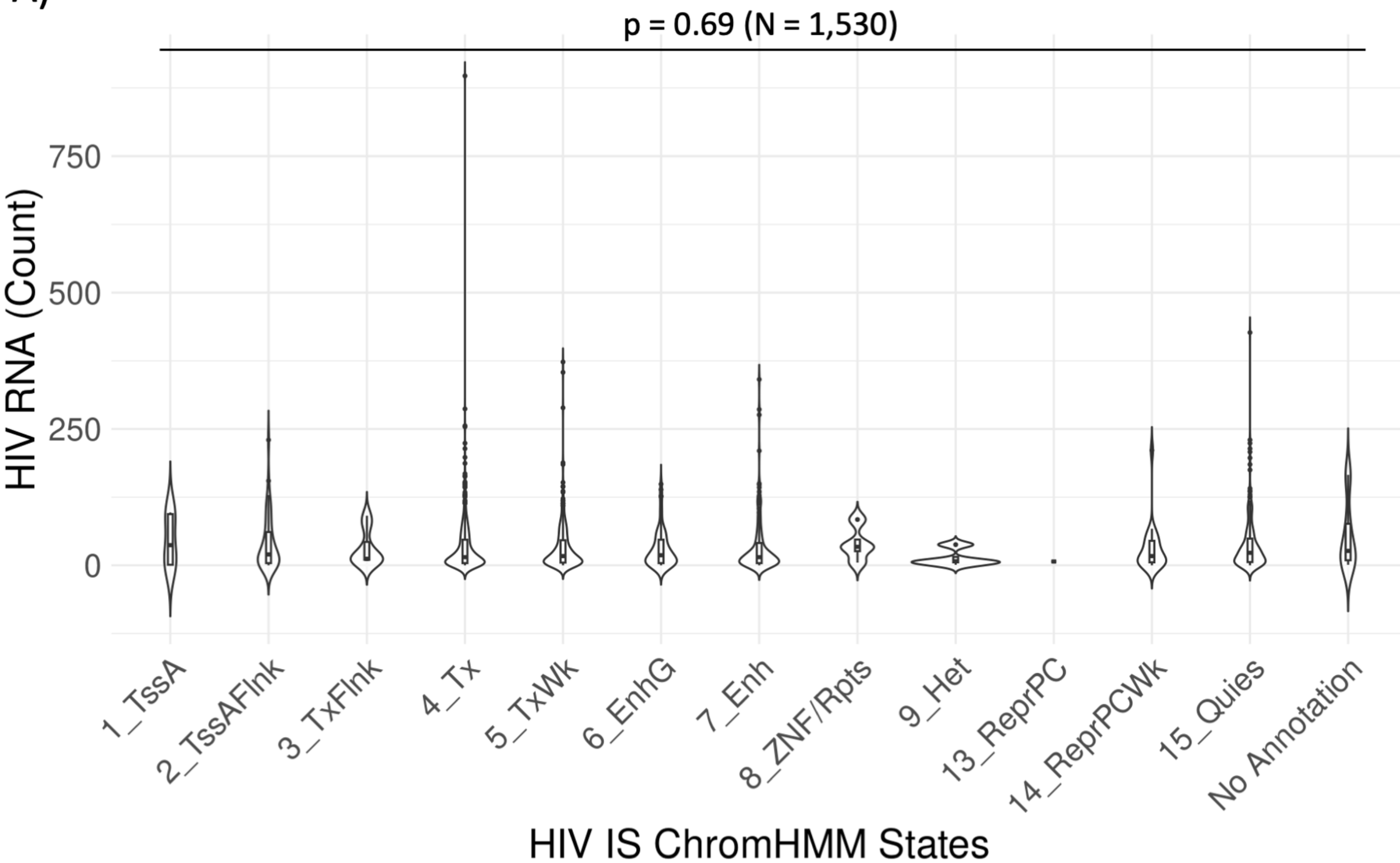

p = 0.69 (N = 1,530)
750
500
250
0
HIV RNA (Count)
1_TssA
2_TssAFlnk
3_TxFlnk
4_Tx
5_TxWk
6_EnhG
7_Enh
8_ZNF/Rpts
9_Het
13_ReprPC
14_ReprPCWk
15_Quies
No Annotation
HIV IS ChromHMM States

B)

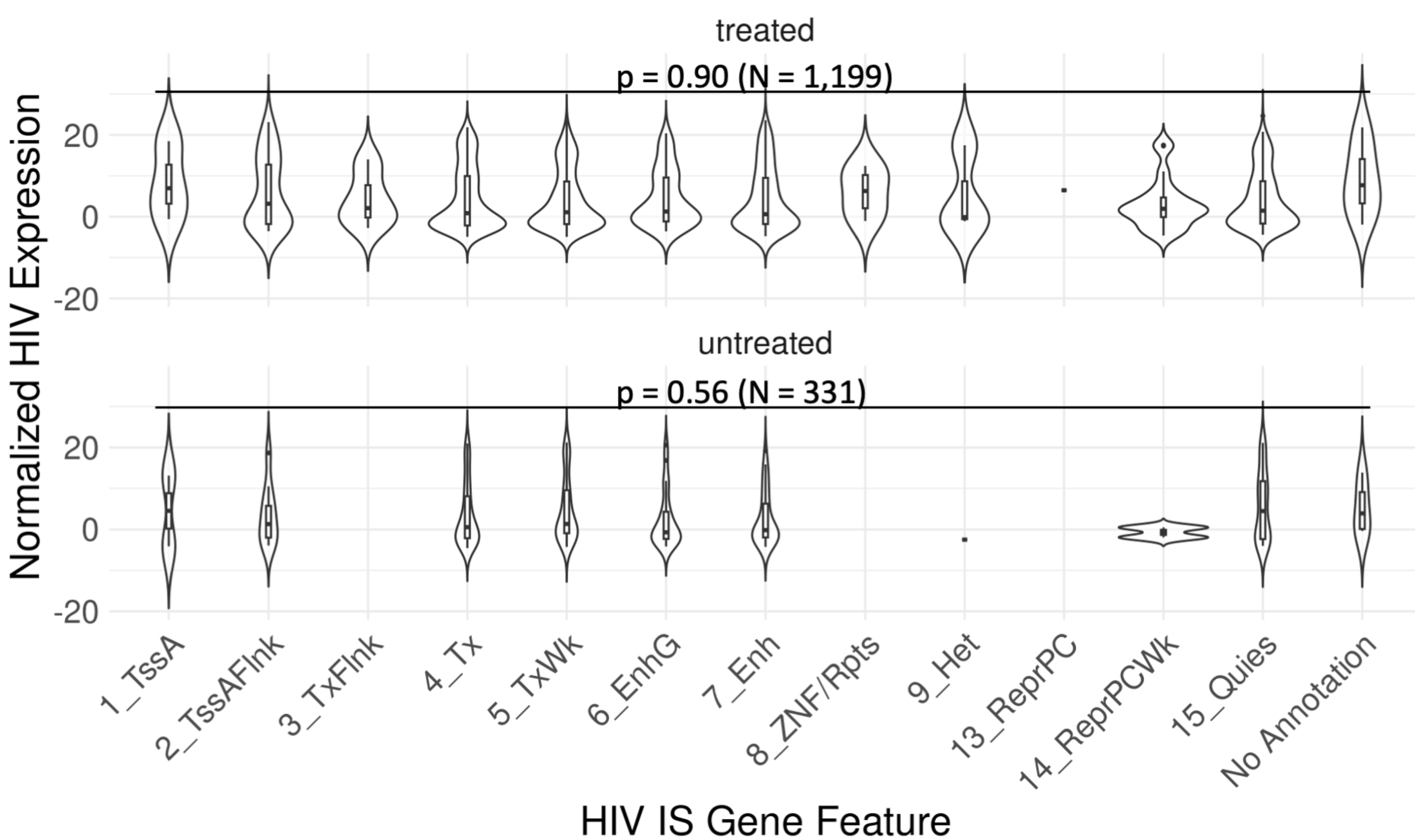

treated
p = 0.90 (N = 1,199)
untreated
p = 0.56 (N = 331)
20
0
-20
Normalized HIV Expression
1_TssA
2_TssAFlnk
3_TxFlnk
4_Tx
5_TxWk
6_EnhG
7_Enh
8_ZNF/Rpts
9_Het
13_ReprPC
14_ReprPCWk
15_Quies
No Annotation
HIV IS Gene Feature

Figure S8

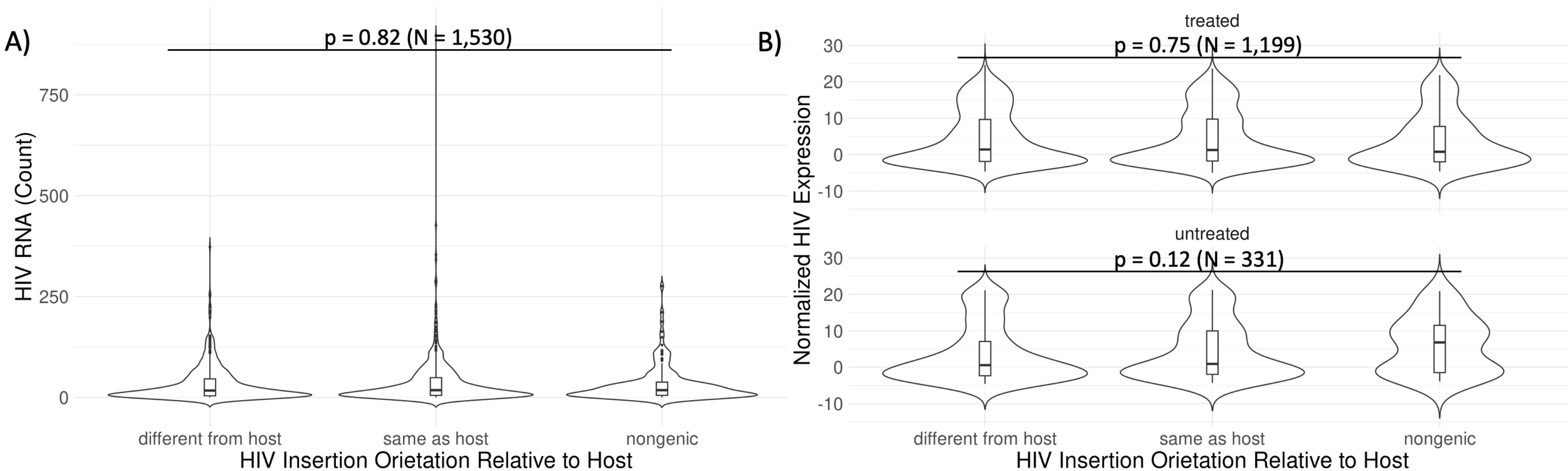
A)
p = 0.82 (N = 1,530)
HIV RNA (Count)
0
250
500
750
different from host
same as host
nongenic
HIV Insertion Orietation Relative to Host
B)
treated
p = 0.75 (N = 1,199)
untreated
p = 0.12 (N = 331)
Normalized HIV Expression
30
20
10
0
-10
different from host
same as host
nongenic
HIV Insertion Orietation Relative to Host

Figure S9

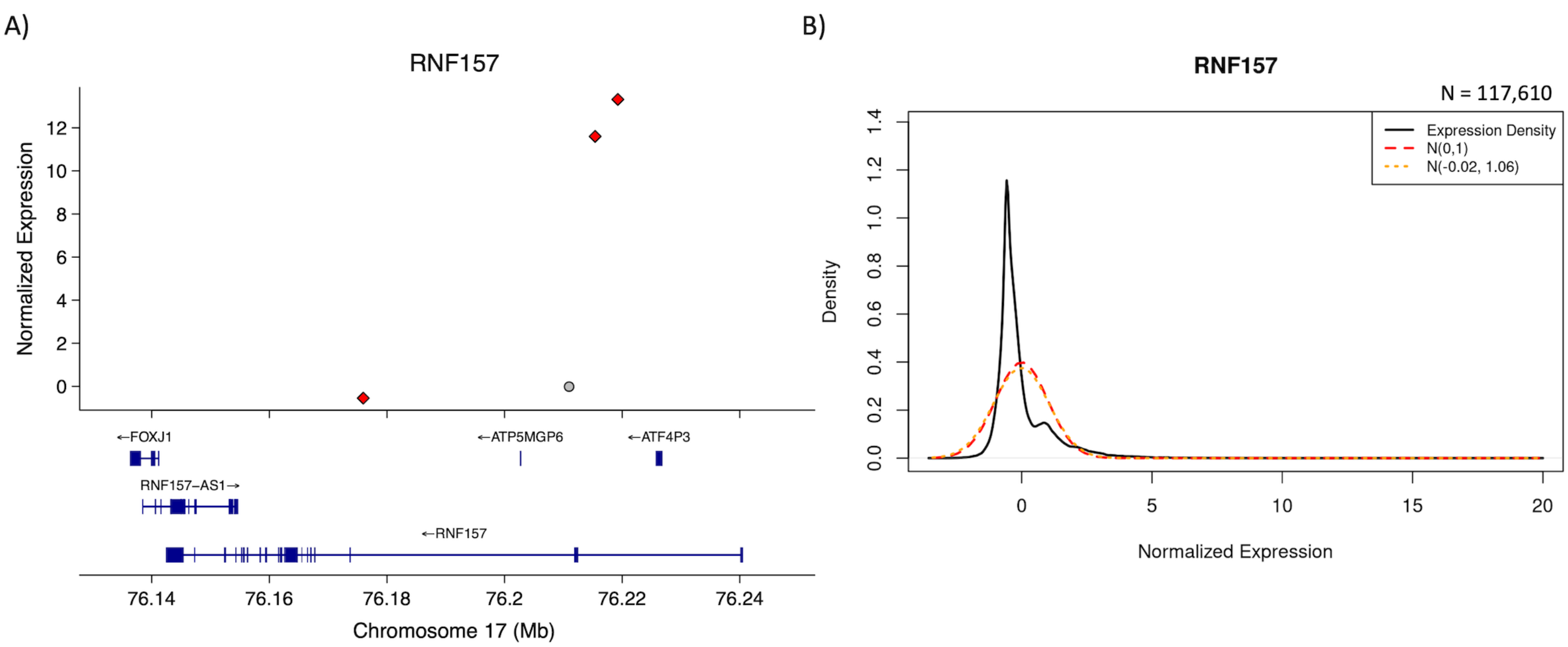
A)
RNF157
Normalized Expression
12
10
8
6
4
2
0
←FOXJ1
←ATP5MGP6
←ATF4P3
RNF157−AS1→
←RNF157
76.14
76.16
76.18
76.2
76.22
76.24
Chromosome 17 (Mb)
B)
RNF157
N = 117,610
Expression Density
N(0,1)
N(-0.02, 1.06)
Density
0.0
0.2
0.4
0.6
0.8
1.0
1.2
1.4
0
5
10
15
20
Normalized Expression

Figure S10

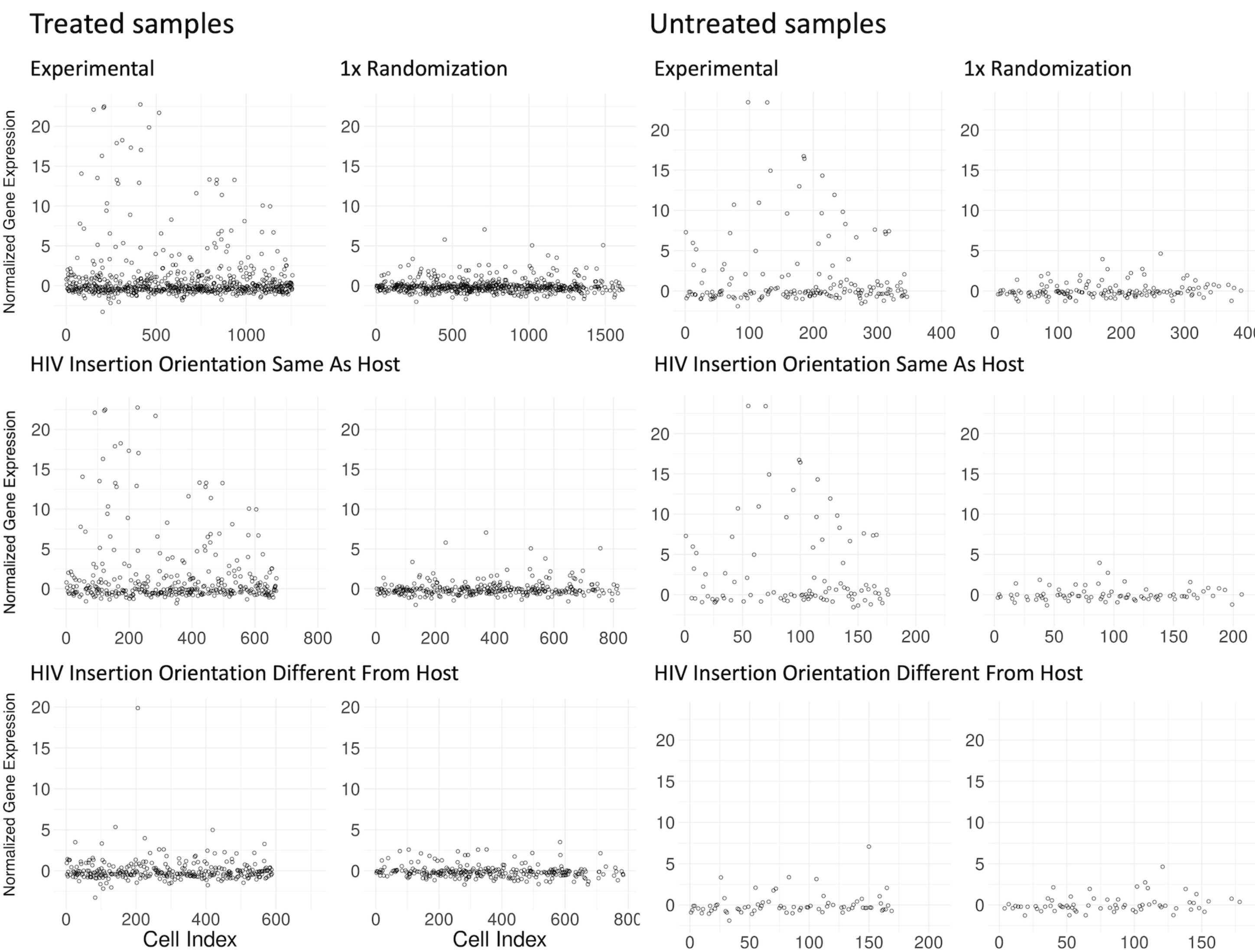
Treated samples
Untreated samples
Experimental
1x Randomization
Experimental
1x Randomization
HIV Insertion Orientation Same As Host
HIV Insertion Orientation Same As Host
HIV Insertion Orientation Different From Host
HIV Insertion Orientation Different From Host
Normalized Gene Expression
Cell Index
