# Supplementary material for: Single-cell analysis of HIV expression and integration sites reveals robust viral expression across diverse chromatin environments
Source: Microbiol Spectr. 2026 Jun 30;14(8):e00670-26. doi: 10.1128/spectrum.00670-26 (PMC13436327; doi:10.1128/spectrum.00670-26)
Supplement: Supplementary file 2 [file spectrum.00670-26-s0002.docx]

**Supplemental Figure 1. Flow cytometry plots of viral gene expression in each sample.** Data showing viral gene expression (GFP) versus side-scatter (SSC) for each sample/condition from each donor (donors 1-5) used for scRNAseq/scATACseq in this study.

**Supplemental Figure 2. Characterization of HIV integration sites (IS). A)** Map of the HIV genome. Each arrow represents a paired chimeric DNA fragment aligned to the HIV genome, with arrow direction and color indicating read orientation. The long terminal repeats (LTRs) are marked by dotted lines. Due to the identical sequences between the 5′ and 3′ LTRs, reads mapping to these regions cannot be distinguished by the aligner and are assigned randomly. However, paired-end sequencing favors short fragments, suggesting that reads mapping deeper into an LTR likely originate from the opposite end of the provirus. This is illustrated in the schematic at the bottom of (**A**). **B**) Pair chimeric rate across chromosomes. The pair chimeric rate is defined as the fraction of chimeric reads relative to total reads for a given chromosome. Blue points represent host–host pair chimerics (one read mapping to one host chromosome, the other to a different host chromosome), while red points represent host–HIV pair chimerics. A recombination prone region on Chromosome 2 and the Y chromosome were excluded.

**Supplemental Figure 3. HIV mRNA distribution. A)** Density plot of total RNA counts per cell, stratified by treatment. **B)** Density plot of unprocessed HIV mRNA counts per cell, stratified by treatment (log-transformed for visualization). Prostratin, iBET151, and SAHA are known latency reversal agents (LRAs). **C)** Stacked bar plot showing the proportion of HIV IS cells in each treatment condition. **D)** Violin and box plots showing the distribution of unprocessed HIV mRNA counts across donors; violins represent density, and box plots indicate the median and interquartile range. **E)** Distribution of HIV mRNA expression after SCTransform normalization and Seurat integration, stratified by donor. **F)** Stacked bar plot showing the proportion of the HIV IS+ cells from each donor. **G)** Density plot of HIV mRNA expression after SCTransform normalization and Seurat integration, stratified by treatment. No units are shown because Seurat integration alters the scale and interpretation of expression values. **H)** Density plot of aggregated SCTransform-normalized and Seurat-integrated HIV mRNA expression across all cells (N = 117,610).

**Supplemental Figure 4. Transcriptomic characterization of differential expression clusters. A)** Heatmap of p-values from pairwise comparisons of total mRNA content per cell across clusters, following the overall significant result shown in Figure 2D. Color intensity indicates statistical significance (Dunn’s test). Due to large sample sizes and imbalance between cluster sizes, Dunn’s test may identify statistically significant but biologically trivial differences. Visual inspection of Figure 2D suggests Cluster 2 as the most distinct. **B)** Heatmaps of p-values from pairwise comparisons of normalized HIV expression per cell across clusters, following the overall significance reported in Figure 2E. **C)** Heatmap displaying the top 10 differentially expressed genes for each cluster. The right panel shows top 5 ontology analysis results for Cluster 2 using Enrichr (32–34). A comprehensive list of DE genes per cluster is provided in the Data Availability section. **D)** Boxplot and violin plot showing unprocessed HIV mRNA counts per cell across clusters, used to validate the trends observed in normalized data (Figure 2E). Kruskal–Wallis tests confirm significant differences across clusters for both IS- and IS+ populations (p < 0.001 for both). Blue indicates IS- cells; orange indicates IS+ cells.

**Supplemental Figure 5. Sample treatment does not influence HIV expression across genic context. A)** Replication of Figure 4B using unprocessed HIV RNA counts. Combined boxplot and violin plot showing the distribution of unprocessed HIV expression levels, categorized by the genic feature (intron, exon, or intergenic) surrounding the HIV integration site. No significant differences were observed (Kruskal–Wallis test, p = 0.95; N = 1,530). **B)** Replication of Figure 4B stratified by small molecule treatment. Combined boxplot and violin plot showing normalized HIV RNA expression by genic feature, separated by treatment condition. No significant differences were observed in either group (p_treated_=0.55, N_treated_ = 1,199; (p_untreated_=0.40, N_untreated_ = 331).

**Supplemental Figure 6. ChromHMM states are generally stable across T cell subtypes.** **A)** Table of 14 T-cell subtypes included in the comparison, PB stands for from Peripheral Blood. Adapted with minor adjustment from Roadmap Epigenomics Consortium et al. 2015 (26) **B)** Transition probability matrix showing the probability of switching from each chromHMM state to all other states across 14 T-cell subtypes from the Roadmap Epigenomics Consortium (26). Values are computed genome-wide, and each row sums to 1. **C)** The same transition probability matrix as in panel A, restricted to genic regions. **D)** Quantification of transition probabilities among states 4–7 within genic regions (results are comparable to those obtained genome-wide, the quantification of all states are available in the data availability section).

**Supplemental Figure 7. Sample treatment does not influence HIV expression across chromatin states. A)** Replication of Figure 5A using unprocessed HIV RNA counts. Combined boxplot and violin plot showing HIV expression across chromatin states. No significant differences were detected (p=0.69, N = 1,530). **B)** Replication of Figure 5A stratified by small molecule treatment. Combined boxplot and violin plot showing normalized HIV RNA expression across chromatin states in treated and untreated groups. No significant differences were observed in either condition (p_treated_=0.90, N_treated_ = 1,199; p_untreated_=0.56, N_untreated_ = 331).

**Supplemental Figure 8. Sample treatment does not influence HIV expression across insertion orientations relative to the host gene. A)** Replication of Figure 6B using unprocessed HIV RNA counts. No significant difference observed across orientations (Kruskal–Wallis test, p = 0.82; N = 1,530). **B)** Replication of Figure 6B stratified by small molecule treatment. Kruskal-Wallis test not significant for either group (p_treated_=0.75, N_treated_ = 1,199; p_untreated_=0.12, N_untreated_ = 331).

**Supplemental Figure 9. Case study shows HIV insertion activation is specific to HIV IS location and orientation relative to the host gene. A)** Scatter plot showing RNF157gene expression in individual cells with HIV integrated into the RNF157 gene. Each point represents a cell; red diamonds indicate HIV in the same transcriptional orientation as RNF157, and gray dots indicate the opposite orientation (52). **B)** Density plot of RNF157 expression across all 117,610 cells after Seurat normalization. The black curve represents the empirical distribution; red and orange curves represent reference normal distributions (mean = 0, SD = 1; and RNF157-specific parameters, respectively). Additional case studies for all 65 insertion-activated genes are available in the Data Availability section.

**Supplemental Figure 10. HIV insertional activation of host gene expression is independent of treatment.** Further stratification of Figure 7A by small molecule treatment condition. Insertional activation remains consistent across treatment groups.

**Supplemental Tables**

**Table S1.**
Sample information, including the number of cells collected for each donor and treatment condition. All donors were seronegative for HIV. SAHA = suberoylanilide hydroxamic acid (Vorinostat), THC = Delta-9-tetrahydrocannabinol.

**Table S2.**
List of all HIV-integrated genes. HIV insertion–activated genes are highlighted in green (threshold: Seurat integrated gene expression ≥ 5). Raw counts for each HIV-integrated gene are also included for reference.

**Table S3.**
Enrichment analysis for HIV insertion–activated genes performed using g:Profiler (31). The second tab includes the search parameters used.

**Table S4.**
Enrichment analysis for HIV insertion–activated genes performed using Enrichr (32–34). Common databases (e.g., GO terms, KEGG, and MSigDB) were included, along with additional databases selected to match those used in Table S3. All significantly enriched terms (adjusted p-value ≤ 0.05) are reported; if no terms were significant, the top five most enriched terms are shown.
